# Wafer-Scale Demonstration of BEOL-Compatible Ambipolar MoS_2_ Devices Enabled by Plasma-Enhanced Atomic Layer Deposition

**DOI:** 10.1021/acsami.5c12014

**Published:** 2025-09-05

**Authors:** Alberto Martínez, Carlos Márquez, Francisco Lorenzo, Francisco Gutiérrez, Manuel Caño-García, Jorge Ávila, José Carlos Galdón Gil, Ruben Ortega Lopez, Carlos Navarro, Luca Donetti, Francisco Gámiz

**Affiliations:** Nanoelectronics Graphene and 2D Materials Laboratory, CITIC-UGR, Department of Electronics, 16741 University of Granada, Granada 18014, Spain

**Keywords:** two-dimensional materials, molybdenum disulfide, atomic layer deposition, TMD transistors, CMOS integration, BEOL compatibility

## Abstract

The relentless scaling of semiconductor technology demands materials beyond silicon to sustain performance improvements. Transition metal dichalcogenides (TMDs), particularly MoS_2_, offer excellent electronic properties; however, achieving scalable and CMOS-compatible fabrication remains a critical challenge. Here, we demonstrate a scalable and BEOL-compatible approach for the direct wafer-scale growth of MoS_2_ devices using plasma-enhanced atomic layer deposition (PE-ALD) at temperatures below 450 °C, fully compliant with CMOS thermal budgets. This method enables the fabrication of MoS_2_-based devices directly on target substrates, eliminating material transfer while ensuring robust adhesion and integration with semiconductor processing. The resulting field-effect transistors (FETs) exhibit stable ambipolar behavior, consistent across semiconductor thickness variations and environmental conditions. Electrical characterization reveals minimal Fermi-level pinning, with Schottky barrier heights below 120 meV for both carriers, supporting a well-defined thermionic transport regime. Low-frequency noise measurements confirm flicker noise characteristics, typical of planar field-effect devices. Material conductivity is significantly enhanced through in situ, BEOL-compatible dielectric passivation or sulfur-atmosphere annealing. This work highlights the potential to directly fabricate, lithographically pattern, and encapsulate MoS_2_ devices for three-dimensional (3D) integration, fully compliant with silicon CMOS thermal constraints.

## Graphical abstract

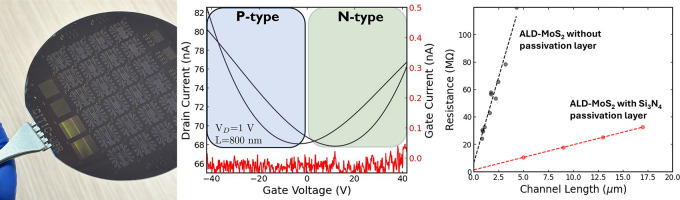

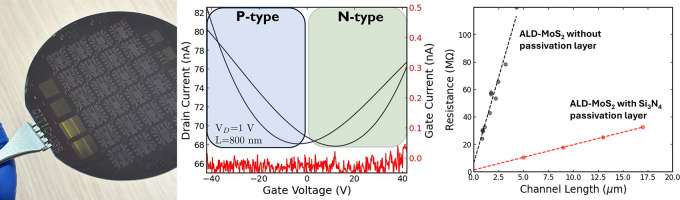

## Introduction

In recent decades, the push for higher transistor densities has driven transformative advancements in electronic devices. As transistor nodes shrink below 2 nm, innovative architectures such as nanosheet, forksheet, and complementary FET (C-FET) designs have been proposed to enhance device performance and overcome the physical limitations of conventional scaling. These architectures exploit the vertical stacking of n-type and p-type MOSFETs to reduce lateral dimensions, requiring the development of novel materials and processes to address challenges such as carrier tunneling and other quantum effects.

Transition metal dichalcogenides (TMDs), a prominent family of two-dimensional (2D) materials, have emerged as promising candidates for next-generation electronic and optoelectronic devices.^1,2^ Their CMOS-compatible bandgaps, large effective masses (which suppress source-to-drain tunneling), and atomic-scale tunability provide distinct advantages for scaling and three-dimensional integration.^3,4^ These properties have already enabled remarkable progress in interconnect scaling for back-end-of-line (BEOL) processes^5^ or 2D barriers and passivation layers.^6^ Moreover, integrating 2D materials into 3D architectures for logic memory cells^7^ and memristors^8^ holds considerable potential for addressing system-on-chip (SoC) challenges. In addition, recent investigations are exploring the influence of different underlying materials on MoS_2_ films deposited by sputtering for the development of 2D-CFETs.^9^

Despite these promising advancements, the integration of TMDs into semiconductor fabrication processes remains challenging.^10,11^ Conventional synthesis methods, including mechanical exfoliation and high-temperature techniques (e.g., chemical vapor deposition at *T* > 450 °C), are not totally suitable for large-scale production or CMOS-compatible workflows.^2^ Moreover, defects and impurities in TMDs often compromise device performance, resulting in phenomena such as Fermi-level pinning, Schottky barrier formation, high contact and sheet resistances, current hysteresis, or Coulomb scattering.^12–15^

A critical challenge lies in effective doping control. Pristine MoS_2_ and WS_2_, two of the most promising TMD materials, theoretically exhibit ambipolar behavior.^16^ However, in practice, they predominantly demonstrate n-type behavior.^17^ Sulfur vacancies, interface states,^16,18–20^ and surface carbon atoms^21^ are key contributors to Fermi-level pinning near the conduction band of the semiconductor.

On the other hand, achieving reliable p-type operation with sulfur as the chalcogen remains a significant challenge.^22^ Success has been achieved in limited cases, such as chemical doping^23^ or through metal-assisted growth techniques.^24^ The presence of adsorbates like H_2_O and O_2_ has also been reported to induce p-type behavior, although these effects are typically removed after material or device annealing.^25^

The experimental demonstration of ambipolar MoS_2_ devices is rare, with reports mainly limited to exfoliated flakes transferred onto PMMA layers^26^ or under ionic liquid gating.^27^ These infrequent occurrences are attributed to substrate-induced Fermi-level pinning, which limits the current flow in any of the branches.^18^

While notable progress has been made in device performance through the use of interfacial insulators (e.g., hexagonal boron nitride),^28^ vertically stacked heterostructures,^29^ and encapsulation techniques, several challenges persist.^10^ These challenges include scalable, wafer-level fabrication, direct growth of 2D materials at CMOS-compatible temperatures for 3D integration, and doping optimization to achieve high-performance p-type and n-type devices using the same material.^18,30,31^

Atomic layer deposition (ALD), a cyclic thin-film deposition technique widely used in the industry for producing thickness-controlled and uniform dielectric films, has recently garnered considerable attention for the synthesis of TMDs.^32^ Plasma-enhanced (PE)-ALD, which employs highly reactive radicals and ionic species, promises to enable reduced growth temperatures and greater flexibility in tailoring the gas-phase chemistry to produce specific film characteristics.^33^ Additionally, PE-ALD often outperforms chemical vapor deposition (CVD) in terms of precise thickness control, film conformality, and uniformity. These aspects make PE-ALD-grown TMDs well-suited to meet the semiconductor industry’s requirements for large-area coverage and low thermal budget synthesis. Notably, MoS_2_-grown at temperatures as low as 100 °C has been demonstrated, allowing precise thickness control and tunable material morphology.^34–36^ Further advancements have enabled chemical doping for p-type devices,^23^ contact optimization,^37^ and front-gate dielectric deposition for CMOS cointegration.^38,39^ PE-ALD-grown MoS_2_ has shown complete wafer coverage with satisfactory uniformity on 200 mm substrates, such as alkali-free glass,^40^ undoped silicate glass, and Al_2_O_3_ wafers.^41^

Despite these advancements, ALD processes typically yield polycrystalline layers with point defects and grain boundaries, often requiring postannealing treatment to enhance crystallinity.^36,37,39,42^ The high temperature of the annealing process typically exceeds the thermal budget constraints. Moreover, challenges such as material delamination during lithography processing or high contact and sheet resistances remain substantial, primarily due to the low adhesion energy of van der Waals layers and the limitations of low-temperature synthesis routes.^38,43^

In this work, we demonstrate the direct, wafer-scale, CMOS-compatible (<450 °C) fabrication of crystalline molybdenum disulfide (MoS_2_) back-gated transistors using plasma-enhanced atomic layer deposition. Devices with varying material thicknesses were systematically characterized both morphologically and electrically.

The low-temperature direct growth on prepatterned pad structures eliminates the need for material transfer, simplifying and accelerating the process. This method enables the formation of crystalline films with ambipolar operation (both p-type and n-type) due to the absence of Fermi level pinning, without requiring postgrowth thermal annealing, ensuring full CMOS compatibility. The ambipolar behavior on transistors offers significant versatility for advanced applications such as reconfigurable devices,^44^ complementary logic, and energy-efficient designs, while also simplifying fabrication by eliminating the need for separate doping or distinct materials. In fact, polarity switching via gate bias transistors, based on black phosphorus, have been demonstrated with promising applications in hardware security circuits.^45^

Furthermore, we provide clear experimental evidence of flicker noise, a characteristic low-frequency noise commonly observed in electronic devices, in these back-gated transistors.

To the best of our knowledge, this is the first report of large-area direct MoS_2_ deposition that consistently exhibits ambipolar behavior across multiple devices fabricated at CMOS-compatible temperatures.

## Experimental Methods

### Substrates Preparation and Devices Photolithography

The material was synthesized at the wafer scale directly on prepatterned source and drain metal electrodes, ensuring better adhesion and minimizing the risk of delamination during subsequent lithographic steps. For the metal stack, ultraviolet (UV) photolithography was employed to define the source and drain patterns directly on the Si/SiO_2_ substrate with a 90 nm thick oxide layer. Both wet etching and lift-off processes were used to define the device pads, with the lift-off process showing superior resolution for submicron channel lengths. Metal evaporation was performed via thermal physical vapor deposition (PVD, Leybold UNIVEX 250) under a vacuum of 8 × 10^–5^ mbar to deposit Cr/Au electrodes (10/90 nm).

Following metal patterning, MoS_2_ was deposited using PE-ALD, as described below. In case of passivation, Si_3_N_4_ encapsulation layer was deposited at 300 °C using bis­(*tert*-butylamino)­silane (BTBAS, SiH_2_(NH*t*Bu)_2_) as the silicon precursor and N_2_ plasma as the coreactant. UV photolithography was repeated to perform a dry etching process that removed the MoS_2_ material outside the channel region between the source and drain contacts. This was achieved using fluorine-based chemistry (SF_6_/Ar: 5/45 sccm) at a power of 50 W and a pressure of 6 mTorr.

### MoS_2_ Synthesis

MoS_2_ deposition was performed using plasma-enhanced atomic layer deposition on an Oxford Instrument FlexAL ALD system, utilizing bis­(*t*-butyl)­(dimethylamido)­molybdenum­(IV) (*Mo*(*N* ^*t*^ *Bu*)_2_(*NMe*_2_)_2_) and hydrogen sulfide (H_2_S) as precursors. The deposition process, summarized in Figure 1, involved the following steps:

1. **Substrate Cleaning:** A remote O_2_ plasma treatment was applied for 1 min to clean the Si/SiO_2_/Cr/Au prepatterned substrate, effectively removing residual organic contaminants.
2. **Precursor Dosing:** *Mo*(*N* ^*t*^ *Bu*)_2_(*NMe*_2_)_2_ was introduced as reactive gas, reacting with the substrate to form a thin chemisorbed layer. The Mo precursor was stored in an electropolished stainless steel bubbler (Pot) at 60 °C to ensure sufficient vapor pressure. The precursor dose was introduced at a pressure of 80 mTorr for 2 s.
3. **Purge Step:** The chamber was purged with Argon gas for 6 s at pressures below 0.1 mTorr (full pump), removing any unreacted precursor molecules and byproducts.
4. **Sulfidation:** Reactive gas (H_2_S/H_2_) was introduced, reacting with the Mo precursor under remote plasma to form MoS_2_. Plasma was activated using 100 W RF power, with a gas flow of 40 sccm Ar, 8 sccm H_2_S, and 2 sccm H_2_ for 30 s, while maintaining a pressure below 0.1 mTorr to sustain the plasma.
5. **Final Purge:** A final Argon purge was conducted for 4 s to eliminate any residual unreacted species and byproducts from the reaction chamber.

This cycle was repeated to achieve the desired MoS_2_ film thickness. The growth temperature inside the chamber was maintained at 400 °C throughout the process.

**Figure 1:**
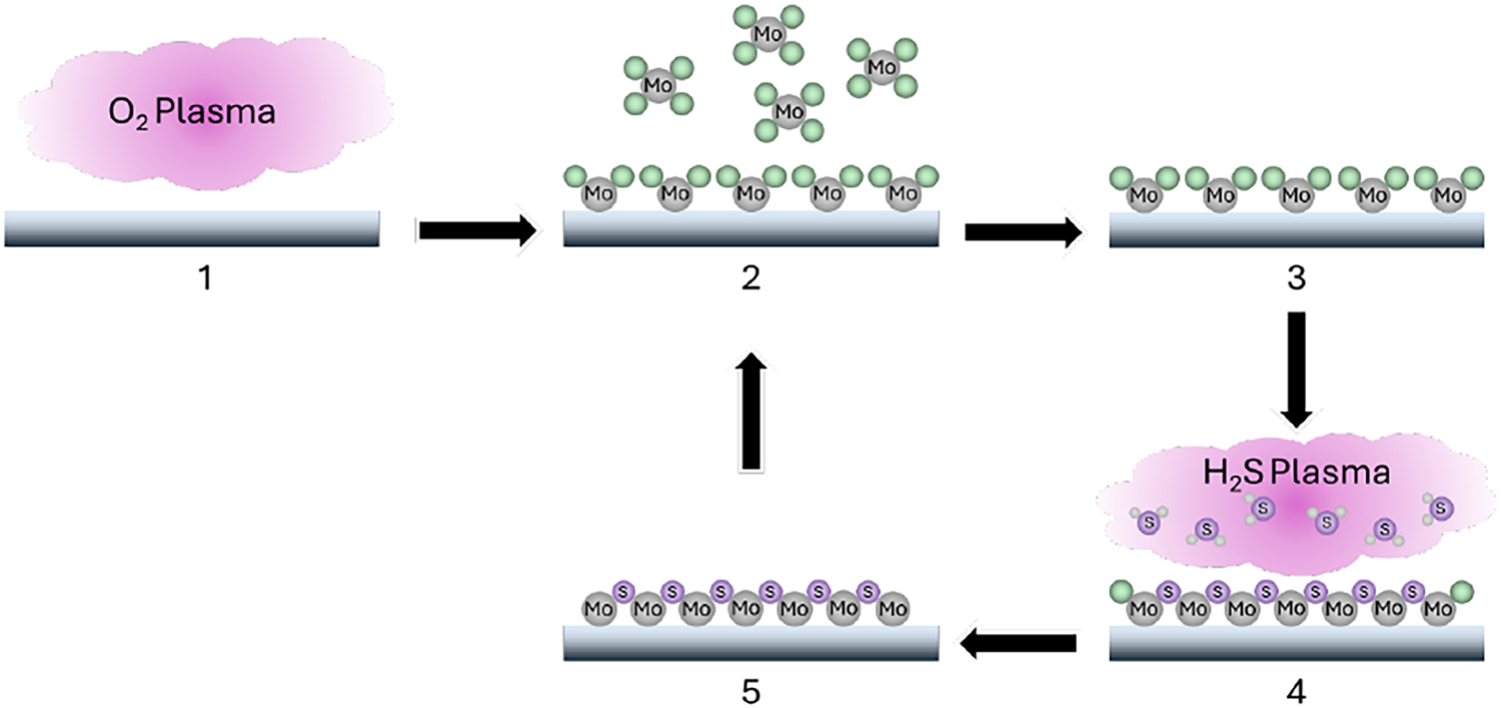
Schematic representation of the plasma-enhanced atomic layer deposition process for MoS_2_.

### Characterization Setup

For structural characterization, atomic force microscopy (AFM), Raman spectroscopy, and X-ray photoelectron spectroscopy (XPS) were employed. AFM was performed using an NTMDT NTEGRA system in semicontact mode with metallic tips. Raman spectra were acquired with a Witec alpha300 system using 532 nm laser excitation, a laser power of 20 mW, a 100× objective, and a grating of 600 lines per mm. Each spectrum was accumulated over 50 scans. XPS measurements were conducted using a Kratos Axis Ultra-DLD spectrometer with Al Kα (1486.6 eV) radiation.

For electrical characterization, a semiconductor analyzer (Keysight B1500) and various temperature- and pressure-controlled probe stations (Suss PA-300PS and Janis cryostat) were utilized. Transfer characteristics (*I*_D_–*V*_G_) were measured using a sweep from −42 to +42 V and back, with 1002 points per curve and a sweep duration of 110 s. A 1-s hold time was applied at the initial gate voltage before the sweep. Low-frequency noise measurements were performed using a low-noise current amplifier connected to a software-based spectrum analyzer.

## Results and Discussion

### Structural and Morphological Characterization

Figure 2a shows a photograph of the as-synthesized samples at the wafer level, highlighting the high MoS_2_ homogeneity and the noticeable color variations corresponding to the different number of ALD cycles employed. Notably, the uniform color of MoS_2_ across the wafer demonstrates its significant homogeneity. Figure 2b presents a directly lithographed wafer featuring various devices and structures prepared for electrical characterization. Different back-gate transistors with varying drain-source lengths and widths, as well as multiple van der Pauw structures and designs for biosensors, were successfully fabricated. Significantly, no material delamination was observed in any of the more than 20 wafers fabricated for this work. Figure 2c illustrates the scanning electron microscopy (SEM) characterization of several devices, where the contacts and TMD material are distinguishable. A zoomed-in atomic force microscopy (AFM) view of a region containing both SiO_2_ and MoS_2_ is provided in Figure 2d. In this etched area, the thickness of the MoS_2_ layer is determined to be around 7 nm. It is important to note that this profile thickness may exceed the actual MoS_2_ layer thickness, as some SiO_2_ substrate may have been etched during the reactive-ion process. Figure S1 shows a zoomed-in area of a MoS_2_ region, corroborating a MoS_2_ layer formed by grains approximately 7 nm in thickness and less than 100 nm in diameter. The MoS_2_ region exhibits an average surface roughness (*R*_a_) of 1.1 nm and a root-mean-square roughness (*R*_q_) of 1.4 nm. This grain size and layer roughness are consistent with the expected results for low-temperature ALD-grown MoS_2_ materials, which exhibit smaller grain sizes compared to the CVD case.^32,38,40,41^

**Figure 2:**
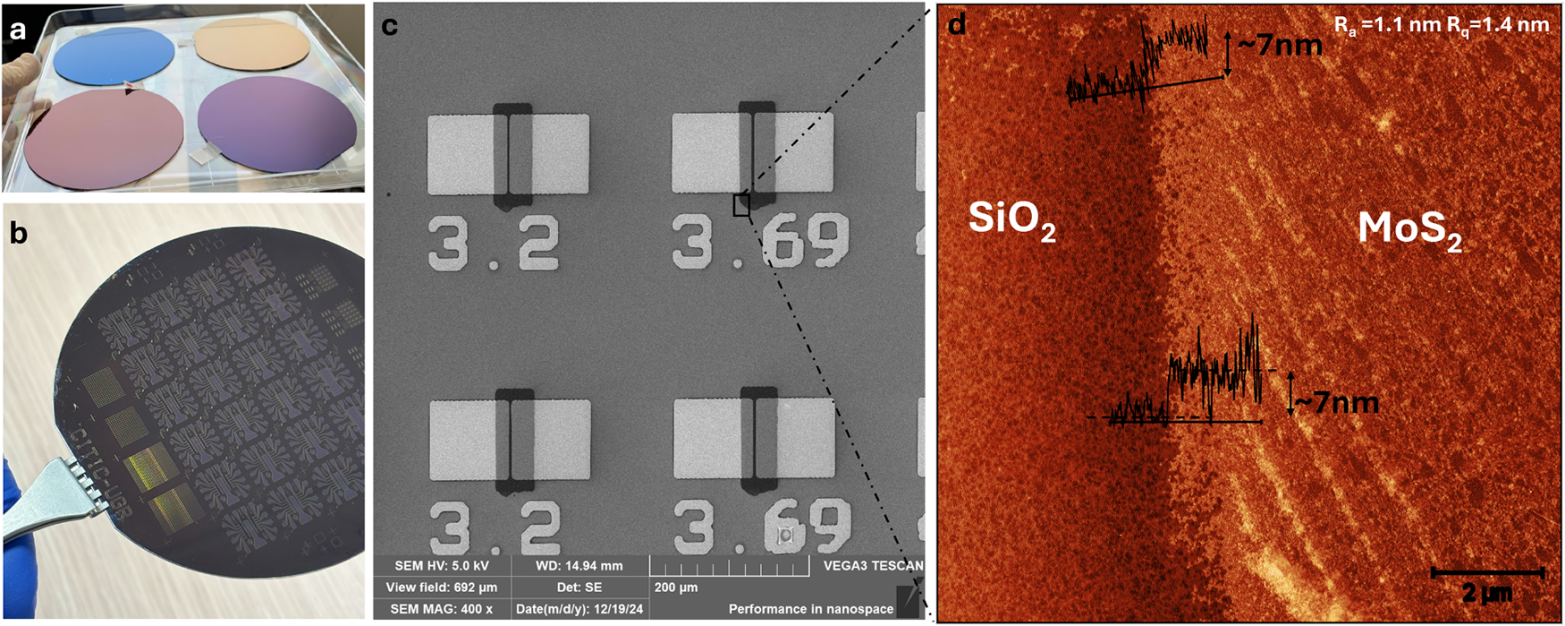
(a) Si/SiO_2_ wafers were coated with MoS_2_ layers of varying thicknesses, deposited using different numbers of cycles in PE-ALD. The synthesis process was conducted at a temperature of 400 °C, with the number of ALD cycles varying from 10 (magenta) to 40 (blue). (b) Photograph of a 100 mm Si/SiO_2_/MoS_2_ wafer after photolithography processing. Various device design layouts, including van der Pauw structures, back-gate transistors, capacitors, and sensors, were fabricated. (c) A scanning electron microscopy (SEM) image showing back-gated devices with varying channel dimensions. Cr/Au was used for the deposition of square pads. (d) Atomic force microscopy (AFM) topography of a SiO_2_/MoS_2_ region, highlighting the distinction between SiO_2_ and MoS_2_ areas. The accompanying profiles indicate a MoS_2_ layer thickness of approximately 7 nm in the etched region for the case of 40 ALD-cycles.

Figure 3a shows the Raman spectrum of the MoS_2_ layer excited by a 532 nm laser under ambient conditions. The spectrum reveals two characteristic Raman bands at 369 and 393 cm^–1^, corresponding to the in-plane (E_2g_^1^) and out-of-plane (A_1g_) vibrational modes, respectively, demonstrating the crystalline nature of the MoS_2_ layer. The peak separation (Δ) is approximately 24 cm^–1^ across the MoS_2_ layer, consistent with multilayer MoS_2_,^46,47^ and also in agreement with the MoS_2_ thickness measured by the AFM topography.

**Figure 3:**
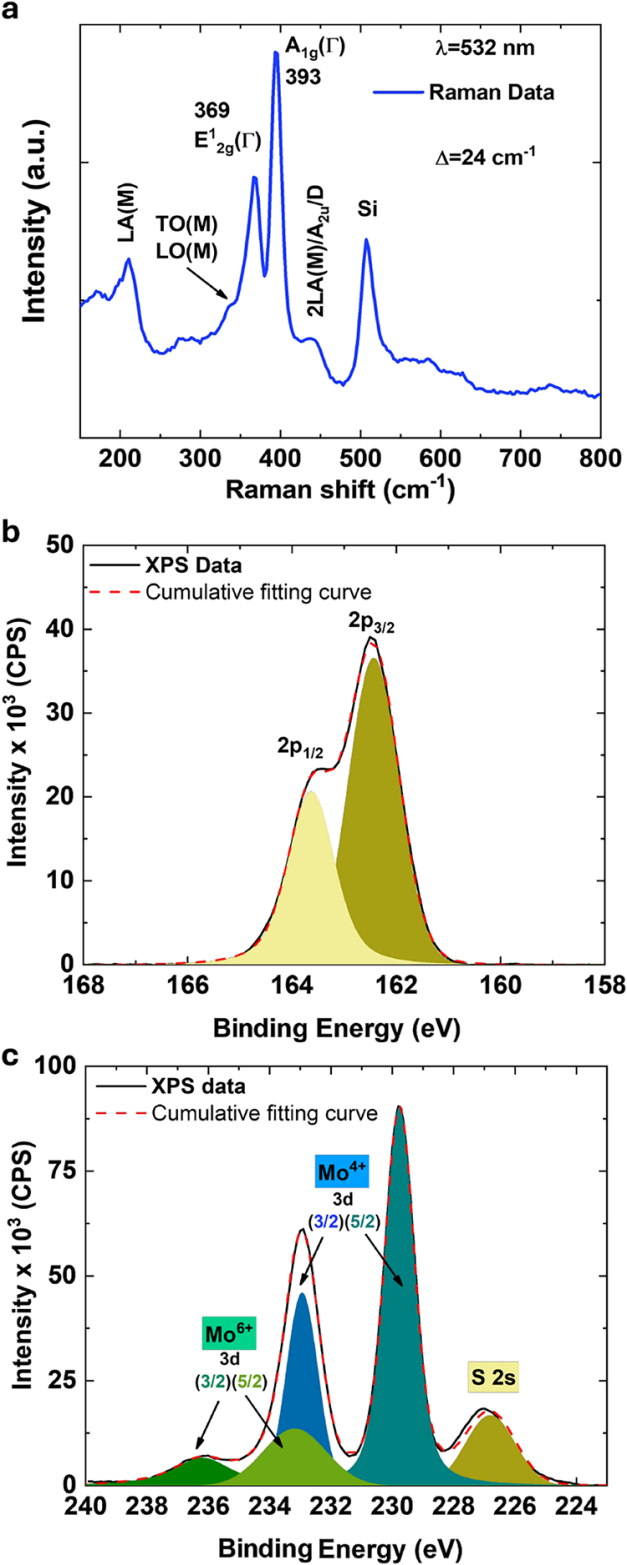
(a) Raman spectrum of the synthesized MoS_2_ under 532 nm light excitation. High-resolution XPS spectrum: (b) S 2p core. (c) Mo 3d core.

Additionally, the Raman spectrum exhibits a peak centered at 440 cm^–1^, which is attributed to a combination of factors:^47^

1. The double frequency of the LA­(M) mode (observed around 210 cm^–1^), which is related to disorder-induced Raman scattering or increased partial oxidation in the MoS_2_ samples.^48^ This phenomenon has also been reported in other ALD-grown MoS_2_ studies.^23,35,40^

2. The first-order optical phonon peak A_2u_, associated with asymmetric translations of Mo and S atoms along the *c*-axis.^49^
3. The D peak, attributed to Mo–S vibrations in oxysulfide species.^50^

These features are further linked to the presence of bridging Mo–S–Mo species in reduced molybdenum compounds or Mo^6+^ oxysulfide species.^47^ Additionally, peaks corresponding to the longitudinal optical (LO) and transverse optical (TO) phonon branches are also observed.^48^ No changes in the Raman characterization were observed before and after the device photolithography process. Figure S2 presents Raman spectra measured across the entire wafer, confirming the previously noted uniformity of the MoS_2_ film.

X-ray photoelectron spectroscopy (XPS) was performed to measure the binding energies of sulfur (S, Figure 3b) and molybdenum (Mo, Figure 3c) atoms. The S 2p peaks at 162.4 and 163.6 eV (Figure 3b) are attributed to the spin–orbit components S 2p_3/2_ and S 2p_1/2_, with a 2p_1/2_:2p_3/2_ ratio of 0.6 (slightly higher than the ideal 0.5 ratio expected for S ions with a single binding state to Mo ions^36^) and a spin–orbit splitting of 1.2 eV. Figure 3c displays two broad peaks at approximately 229.7 and 232.9 eV, corresponding to the main doublet Mo 3d_5/2_ and Mo 3d_3/2_ of the Mo^4+^ chemical state, with a 3d_3/2_:3d_5/2_ ratio of 0.46 and a spin–orbit splitting of 3.1 eV.^51,52^ Additionally, a minor peak at 226.8 eV is assigned to the S 2s component. These results, along with the wide XPS spectrum in Figure S3a, are consistent with previously reported binding energy values for MoS_2_.^53^ Smaller contributions at approximately 233.2 and 236.2 eV correspond to binding energies typically attributed to Mo^6+^ oxidation states, indicating the presence of suboxide species.^52^ The Mo^6+^ peaks suggest oxygen incorporation into the synthesized MoS_2_ film,^52,54^ which is corroborated by the Raman results mentioned earlier and the Mo–O peak observed in the O 1s spectrum of Figure S3b. These findings imply the presence of defects that distort the stoichiometry of the MoS_2_ films, consistent with observations in other ALD-grown MoS_2_ samples under similar growth conditions.^35,36^ However, Mo^5+^ states, which are also commonly associated with oxide incorporation in CVD-grown MoS_2_,^19^ are not detected in these samples.

### Electrical Characterization

The output (*I*_D_–*V*_D_) and transfer (*I*_D_–*V*_G_) characteristics of back-gated devices with varying channel lengths (*L*) and a fixed channel width (*W*) of 100 μm are presented in Figure 4a,b, respectively. The output characteristics exhibit linear behavior and symmetrical current responses for both positive and negative voltages, indicative of low Schottky barrier contacts.^19,20^ Furthermore, as the channel length decreases, the output current increases, consistent with channel-limited transport. This behavior contrasts with the contact-limited injection observed in the p-branch of other ALD-grown devices.^38^

**Figure 4:**
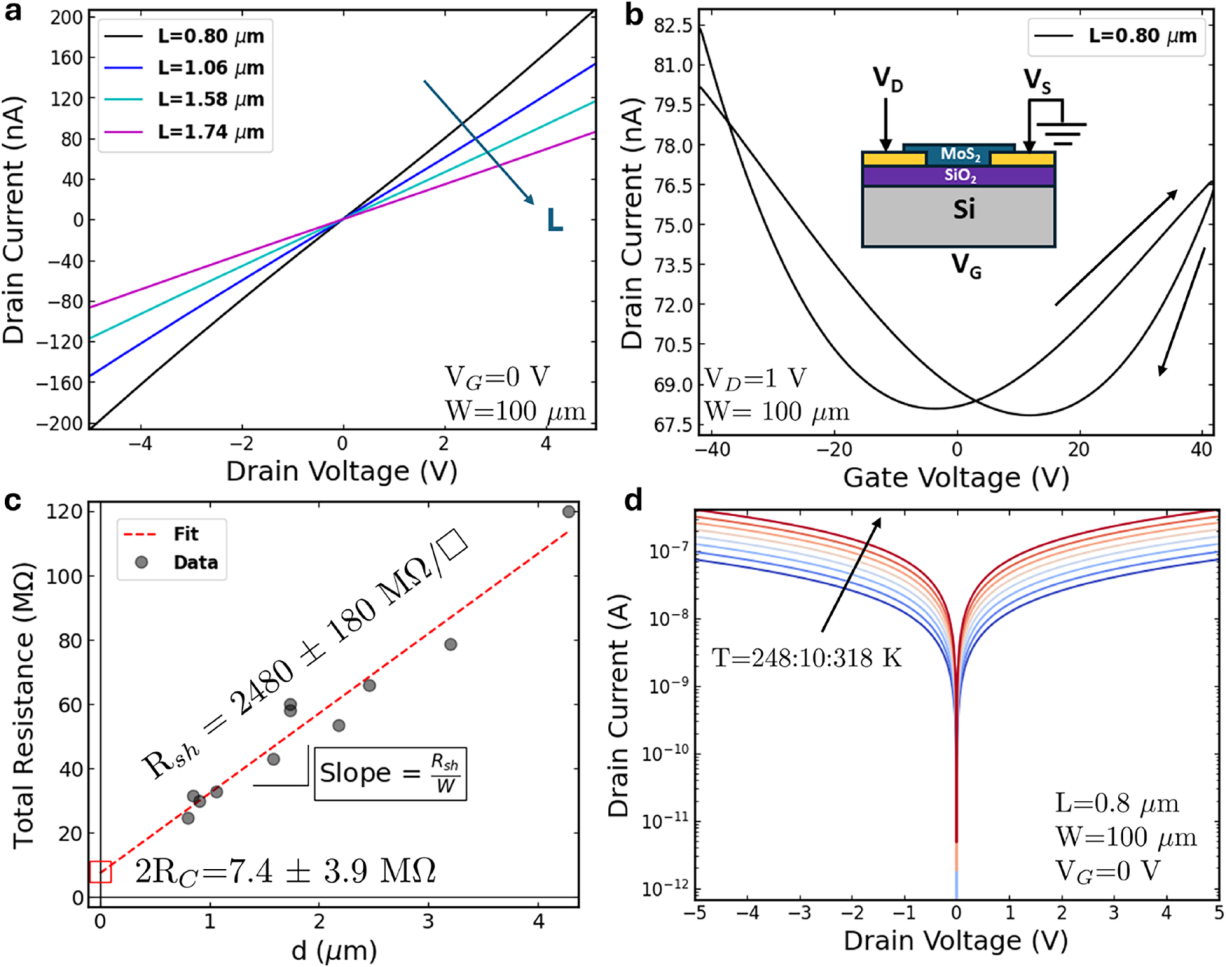
(a) Drain current versus drain voltage characteristic for devices with *W* = 100 μm and varying channel lengths (*L*). (b) Transfer characteristics (*I*_D_–*V*_G_) for a device with *L* = 800 nm. The experimental setup and device cross-section are shown in the inset. (c) Extrapolation of contact resistance (*R*_c_) and sheet resistance (*R*_sh_) for a series of devices with different contact distances or pitch (*d*) and *W* = 100 μm. (d) Output characteristics (*I*_D_–*V*_D_) at various temperatures ranging from 248 to 318 K.

The transfer characteristics (*I*_D_–*V*_G_) measured in the devices, following the schematic shown in the inset of Figure 4b, demonstrate the modulation of channel resistance with the applied back-gate voltage. The double-swept curves exhibit hysteresis, which is commonly attributed to charge trapping and detrapping phenomena associated with defects.^55^ The back-gate current (Figure S4) remains significantly lower than 100 pA, indicating that gate leakage does not substantially affect the observed behavior. Notably, these devices exhibit clear ambipolar performance, with both hole (negative gate voltages) and electron (positive gate voltages) conduction. As introduced, such behavior is rare in MoS_2_ devices, regardless of the synthesis method, where n-type characteristics are predominantly observed.^56,57^

The ambipolar behavior here reported was consistently observed across all measured devices, regardless of the number of ALD cycles (ranging from 20 to 60) (Figure S5) and applied drain voltages (Figure S6).

Despite this promising behavior, certain limitations, such as a low on/off current ratio and low current levels, are observed. These performance constraints may be related to several factors, including Schottky barriers, carrier population limitations due to defect trapping effects, reduced current modulation with back-gate voltage in thick materials, or Fermi-level pinning at the SiO_2_ substrate.^18^

To further elucidate these phenomena, the Transmission Line Model (TLM) was employed to extract both the sheet resistance and contact resistance of the devices.^58^ Figure 4c shows the total resistance as a function of contact separation distance (*d*) at zero back-gate voltage. From this analysis, the *y*-intercept yields the total contact resistance (2*R*_c_ ≈ 7.4 MΩ), while the slope corresponds to the sheet resistance of the MoS_2_ channel (2480 MΩ/□). The dependence of the resistance on the full range of gate voltages is presented in Figure S7.

These results were corroborated using Four-Contact characterization performed on the as-synthesized materials.^58^ Devices synthesized at two different temperatures (350 and 400 °C) were characterized for varying numbers of ALD cycles (Figure S8). A clear trend of decreasing sheet resistance with increasing ALD cycles and lower synthesis temperatures was observed. It is important to note that material resistivity typically decreases with lower synthesis temperatures, although this often comes at the expense of crystallinity.^40^ In this case, sheet resistances as low as 130 MΩ/□ were achieved, values in good agreement with those reported for ALD-grown WS_2_ ^31^ and MoS_2_ devices.^23^ Considering the AFM-measured thicknesses, the corresponding resistivity (ρ) is approximately 10^2^ Ω cm for as-synthesized samples with 75 ALD cycles at 350 °C.

Figure 4d illustrates the expected increase in current levels at elevated temperatures, according to the typical behavior of semiconductors. Symmetry between positive and negative *V*_D_ voltages is still observed. This temperature-dependent characterization is first employed to investigate the Schottky barrier height at the metal/semiconductor junction. In the thermionic emission regime, the electrical transport is described by^16,59,60^

1 $$I_{D}=AA^{*}T^{2}e^{-\left(q/k_{B}T\right)\left(\phi_{B}-\left(V_{D}/n\right)\right)}$$

where *A* is the diode area, A* is the Richardson constant, *q* is the elementary charge, *k*_B_ is the Boltzmann constant, ϕ_B_ is the Schottky barrier height, *n* is the ideality factor, and *T* is the temperature. The slope *S*(*V*_D_) is obtained by extrapolating ln­(*I*_D_/*T* ^2^) versus 1000/*T* for different *V*_D_.

Figure 5a shows the Arrhenius plot for a device with a length of 800 nm, width of 100 μm, and varying drain voltages. The Schottky barrier height is obtained from the extrapolated slope at zero *V*_D_ (S_0_)­

2 $$S_{0}=-\frac{q\Phi_{b}}{1000k_{B}}$$

**Figure 5:**
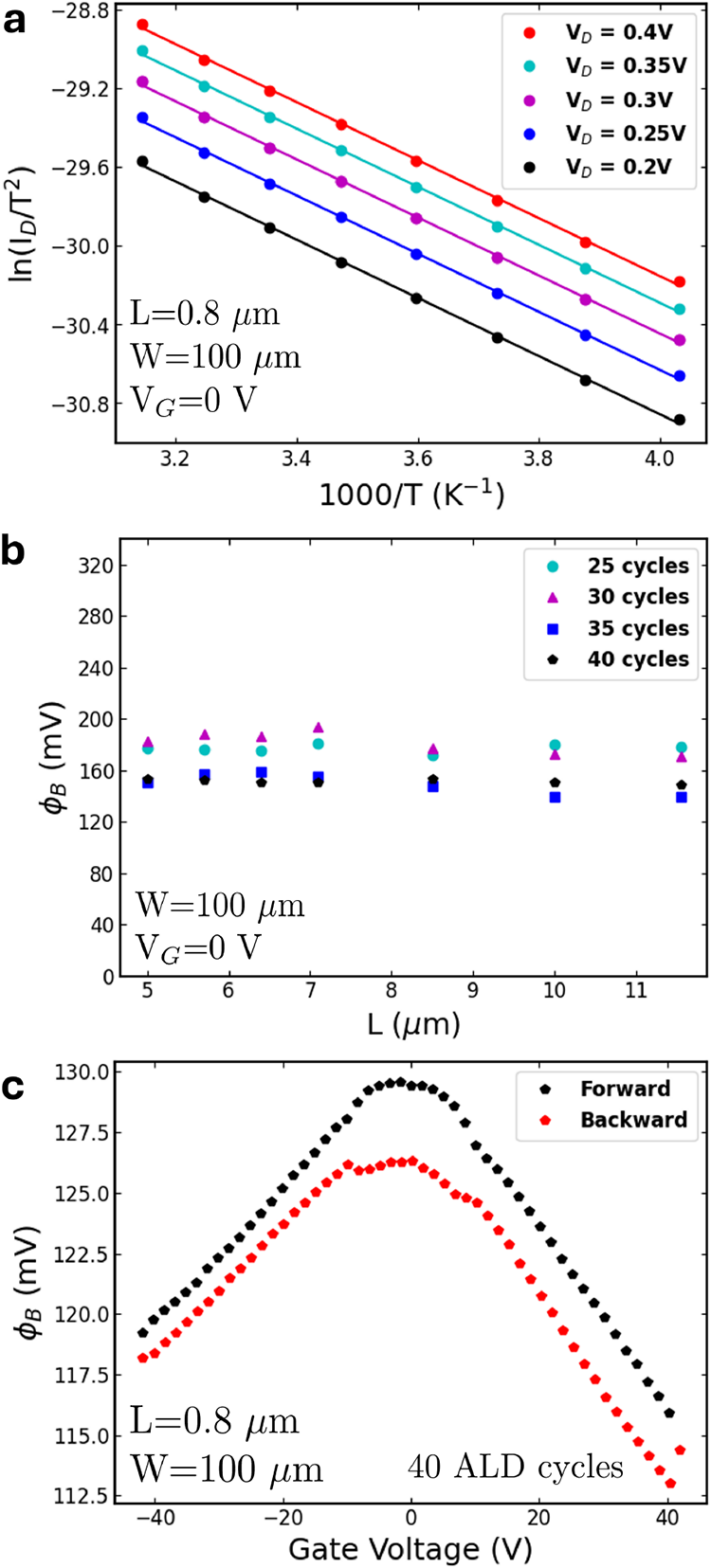
(a) Arrhenius plot showing ln­(*I*_D_/*T* ^2^) versus 1000/*T* curves for various *V*_D_ values for a device with *L* = 0.85 μm and *W* = 100 μm. (b) Extrapolated Φ_B_ for devices with different numbers of ALD cycles and varying channel lengths. (c) Extrapolated Φ_B_ as a function of gate voltage. Unless otherwise indicated, the samples were synthesized with 40 ALD cycles.

Figure 5b presents the Schottky barrier heights extrapolated for devices with different channel lengths and varying numbers of ALD cycles, measured at zero gate voltage. Values ranging from 150 to 200 mV are observed across all cases. Devices with a lower number of ALD cycles exhibit higher Schottky barrier heights, suggesting a correlation with layer thickness. The Schottky barrier height presents no dependence on the device length. Figure 5c illustrates the Φ_B_–V_G_ relationship, which shows a linear and decreasing dependence on *V*_G_ for both electron and hole branches. Slight differences between forward and backward bias sweeps are observed. Same results in other MoS_2_ thicknesses have been shown in Figure S9.

The observed symmetry in the Schottky barriers for electrons and holes is consistent with the ambipolar behavior and, consequently, with the absence of strong Fermi-level pinning. However, the relatively low extracted barrier heights are difficult to attribute solely to gate-induced band bending and thermionic emission, especially considering the reported bandgap for MoS_2_ (1.29–1.90 eV).^61^ This discrepancy suggests that additional mechanisms may significantly influence the effective barrier height.

To gain further insight into the origin of this behavior, we independently analyzed the temperature dependence of the contact resistance (*R*_c_) and sheet resistance (*R*_sh_), as detailed in Supporting Note 1 (Figures S10–S16). Sheet conductivity is consistent with a thermally activated nearest-neighbor hopping conduction mechanism, which is typically indicative of charge transport through localized states. Although the MoS_2_ films are grown in a crystalline phase, structural disorder (possibly arising from chalcogen vacancies, grain boundaries, or other point defects) may introduce localized states within the bandgap. These defects may cause band tailing and bandgap narrowing.^62^ This interpretation is supported by the temperature dependence of *R*_c_, which aligns with the thermionic emission model^59^ and yields Schottky barriers consistent with the previously extracted in Figure 5.

Another relevant parameter is the field-effect mobility (μ_FE_), which is given by

3 $$\mu_{\mathrm{FE}}=\frac{L}{WC_{\mathrm{ox}}V_{D}}\cdot \frac{dI_{D}}{dV_{G}}$$

where *C*_ox_ is the oxide capacitance per unit area. Figure 6a shows the μ–*V*_G_ curves on a logarithmic scale for different temperatures. Linear dependence of μ on *V*_G_ is evident over a certain range, with a continuous increase in mobility. This *V*_G_-dependent mobility has been previously documented in 2D materials and can be explained by two effects: First, as carrier concentration increases, screening gets stronger, reducing the scattering potential and thus increasing the Coulomb impurity-limited mobility.^63^ Second, as *V*_G_ increases, more trap states in the material are filled, allowing more carriers to remain free to move, which further enhances mobility.^64^ In all cases, the mobility values are consistent with those reported for ALD-grown 2D materials in the literature,^23,37,43^ as confirmed by benchmarking presented in the Supporting Information (Tables S1 and S2). The dependence of mobility on temperature, as depicted in Figure 6b, aligns with a trap-limited and charged-impurity Coulomb scattering mobility regimes^63,65^ which can be in agreement with thermally activated hopping transport mechanism. The low mobility indicates a high density of trap states, which also contribute to the hysteretic behavior observed in the transfer characteristic shown in Figure 4b. This hysteresis arises from the trapping and detrapping of charge carriers, whose potential adds to that of the back-gate.^13,19,66^

**Figure 6:**
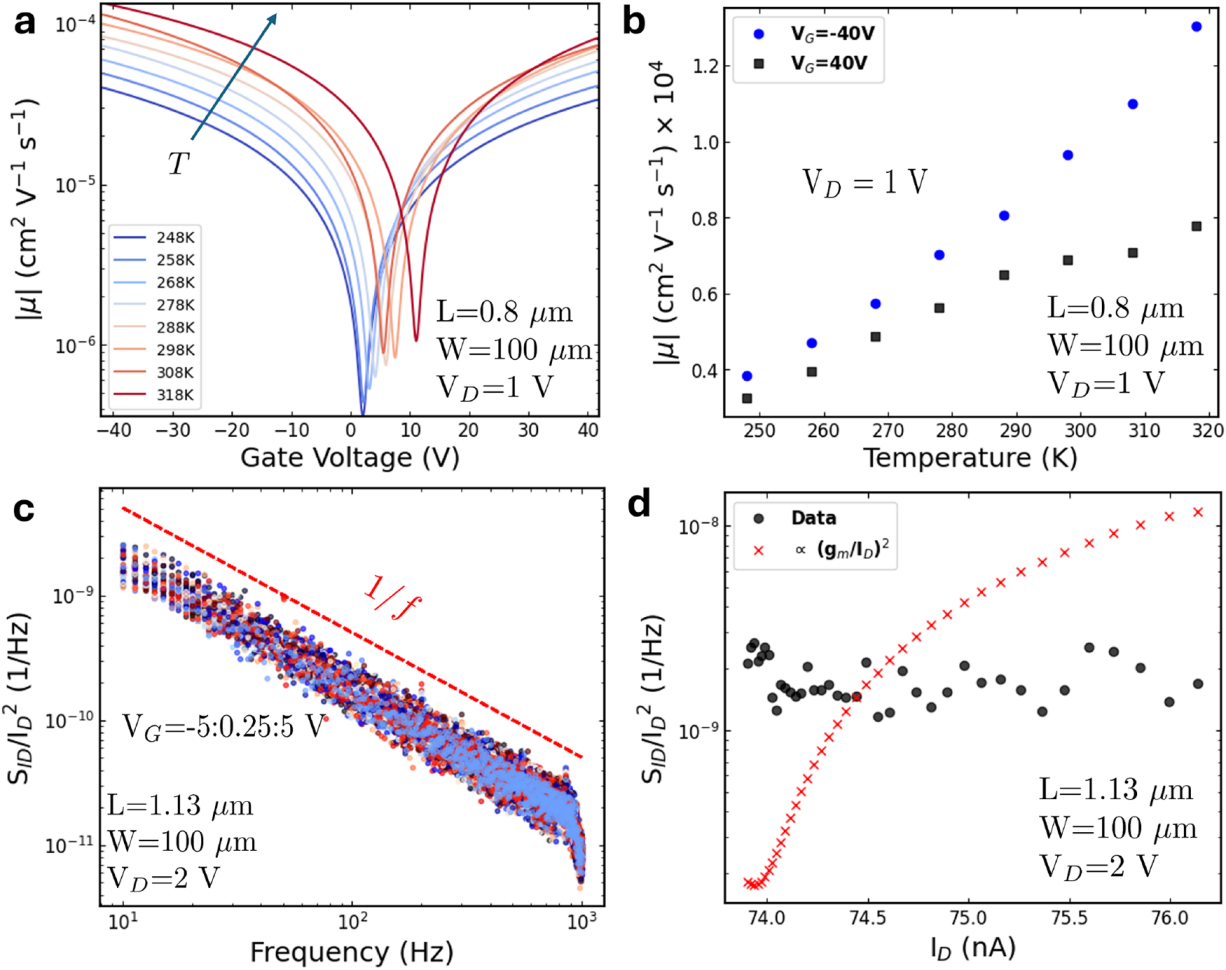
(a) Field effect mobility versus temperature for a device with *L* = 0.8 μm and *W* = 100 μm. (b) Modulation of mobility with temperature. (c) Normalized low-frequency noise spectra of a device with *L* = 1.13 μm and *W* = 100 μm at different back-gate voltages ranging from −5 to +5 V, showing a clear 1/*f* characteristic. The device is operated in the ON state with a source-to-drain bias of 2 V. (d) Normalized spectral noise (black circles) and ∝ (*g*_m_/*I*_D_)^2^ fit versus drain current obtained at different gate voltages.

Another critical parameter, essential for benchmarking new devices, is the low-frequency noise (LFN). The normalized noise spectrum for various back-gate biases is shown in Figure 6c. As depicted, the device exhibits 1/*f* noise characteristics, demonstrating a flicker noise due to charge carrier trapping and detrapping at defect states or impurities in the material.^67^

Figure 6d presents the normalized device noise as a function of the drain current for varying gate voltages. The noise shows minimal dependence on the drain current, ruling out Hooge mobility fluctuations within this voltage–current range.^68^ Similarly, carrier number fluctuations are excluded, as the noise remains flat even when (*g*_m_/*I*_D_)^2^ increases.^69^ As detailed in Supporting Note 2, the data are fitted using a combined carrier number fluctuation and correlated mobility fluctuation (CNF-CMF) model, yielding a surface trap state density on the order of 10^13^ cm^–2^ eV^–1^ (Figure S17). However, the constant normalized noise level observed across devices with varying channel lengths suggests significant resistance fluctuations at the metal–semiconductor interface (Figure S18), indicating a contact-dominated regime within these voltage ranges.^70^

To investigate whether thermal annealing affects one of the carrier branches (holes or electrons) or the material resistivity, annealing processes were performed at a maximum temperature of 700 °C for 15 min in both sulfur-rich and inert gas environments at low pressure (3 Torr). Figure 7 shows that sulfur-rich annealing increases the current levels (decreases material resistivity) while partially suppressing the n-type branch, in line with sulfur vacancy suppression.^40^ In contrast, inert gas annealing results in only a slight improvement in the current levels. Notably, the p-type branch remains unaffected by the annealing process, with current levels increasing, thereby demonstrating persistent ambipolar behavior and ruling out any influence from chemi-absorbed impurities such as O_2_ or H_2_O.

**Figure 7:**
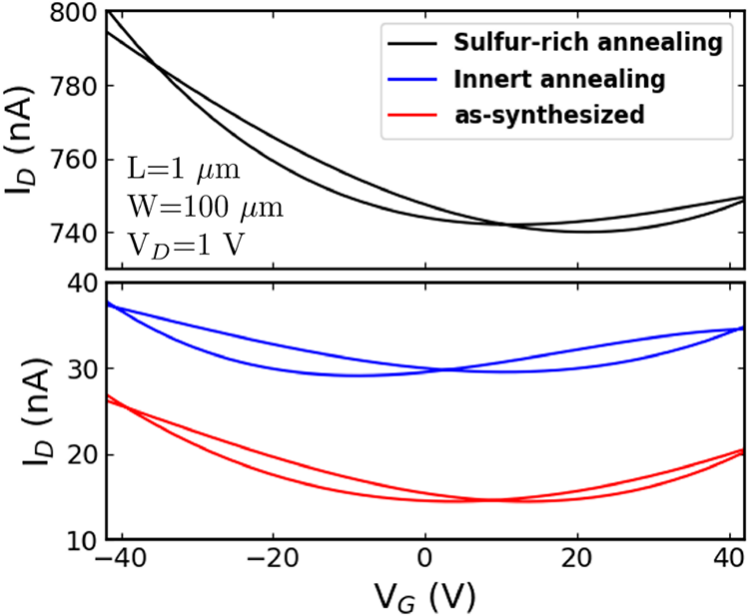
Transfer characteristics of MoS_2_ devices before and after sulfur-rich and inert annealing processes carried out at 700 °C and low pressure for 15 min.

The preservation of ambipolar behavior, regardless of ambient-related effects, was further corroborated using a complementary passivation approach. A thin Si_3_N_4_ layer was sequentially deposited by ALD after MoS_2_ growth, avoiding any exposure to ambient conditions. As shown in Figure S19a, the passivated devices retained their ambipolar characteristics, confirming that the behavior is intrinsic and not dominated by surface adsorbates. At the same time, the devices exhibited enhanced performance, including a consistent reduction of sheet resistance by at least 1 order of magnitude (Figure S19b) and a noticeable decrease in hysteresis, suggesting improved material quality and reduced trapping effects due to surface protection. Additionally, artificial light exposure and vacuum ambient conditions were evaluated during the electrical characterization of the devices, revealing only slight variations in the current levels, as shown in Figure S20.

## Conclusions

This work demonstrates the direct wafer-scale fabrication of ambipolar MoS_2_ back-gated FETs at BEOL-compatible temperatures using plasma-enhanced ALD. The devices exhibit stable ambipolar behavior, low Schottky barrier heights, and minimal Fermi-level pinning, enabling efficient operation in both n- and p-type regimes. An extensive transport analysis reveals thermionic emission as the dominant mechanism, with structural disorder and defect-induced states facilitating balanced electron and hole injection.

While these results establish the potential of MoS_2_-based devices for 3D integration within silicon CMOS technology, further improvements are needed to enhance performance and integration. Ongoing efforts targeting defect passivation through in situ, BEOL-compatible dielectric encapsulation or sulfur-rich annealing have demonstrated promising results improving material conductivity. Additionally, engineering of gate dielectric and contact interfaces is expected to reduce interface trap densities and contact resistance, enabling higher mobility and better switching characteristics. Combined with scalable device patterning, these strategies will help realize energy-efficient, reconfigurable logic circuits and advanced CMOS-compatible architectures. The insights and methodologies presented here provide a strong foundation for the continued advancement of 2D-material-based electronics.

## Supplementary Material



## Notes

AFM topography of MoS_2_ grains; Raman wafer mapping and average spectra; XPS survey and O-1s spectra; transfer characteristics and leakage currents; device statistics and Neutral Charge Point trends; output/transfer curves at varied biases; contact and sheet resistance vs gate voltage; sheet resistance and current vs synthesis conditions; temperature-dependent transfer and Schottky barrier data; transport mechanism analysis; ALD-grown TMD device benchmarking; low-frequency noise and trap density; in situ dielectric passivation improvements; light/dark and air/vacuum device responses (PDF)
