# Supplementary material for: Wafer-Scale Demonstration of BEOL-Compatible Ambipolar MoS2 Devices Enabled by Plasma-Enhanced Atomic Layer Deposition
Source: ACS Appl Mater Interfaces. 2025 Sep 5;17(37):52902–12. doi: 10.1021/acsami.5c12014 (PMC12447386; doi:10.1021/acsami.5c12014)
Supplement: Supplementary file 1 [file am5c12014_si_001.pdf]

Supporting Information

Wafer-Scale Demonstration of BEOL-Compatible Ambipolar MoS_2 Devices Enabled by Plasma-Enhanced Atomic Layer Deposition

Alberto Martínez[†], Carlos Márquez[†]*, Francisco Lorenzo, Francisco Gutiérrez, Manuel Caño-García, Jorge Ávila, José Carlos Galdón Gil, Ruben Ortega Lopez, Carlos Navarro, Luca Donetti and Francisco Gámiz

Nanoelectronics, Graphene, and 2D Materials Laboratory, CITIC-UGR, Department of Electronics, University of Granada, Granada, 18014, Spain
[†] These authors contributed equally to this work.

*Corresponding author: carlosmg@ugr.es

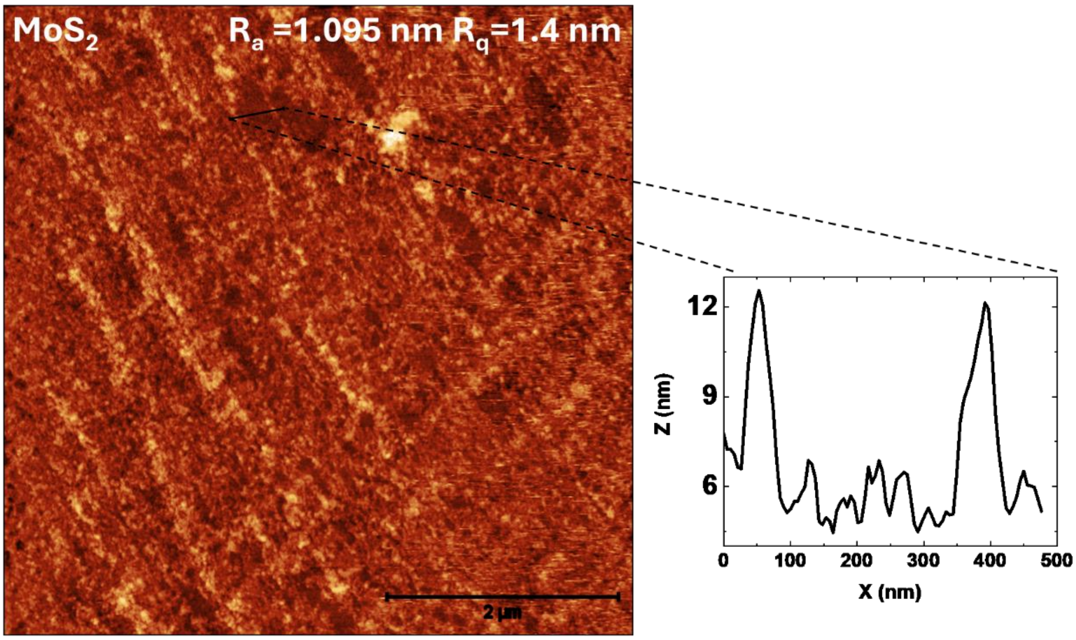

Figure S1. Atomic force microscopy (AFM) topography of a MoS_2 region. The included profile in an area with breaks indicates a MoS_2 layer formed by grains approximately 7nm in thickness and less than 100nm in diameter.

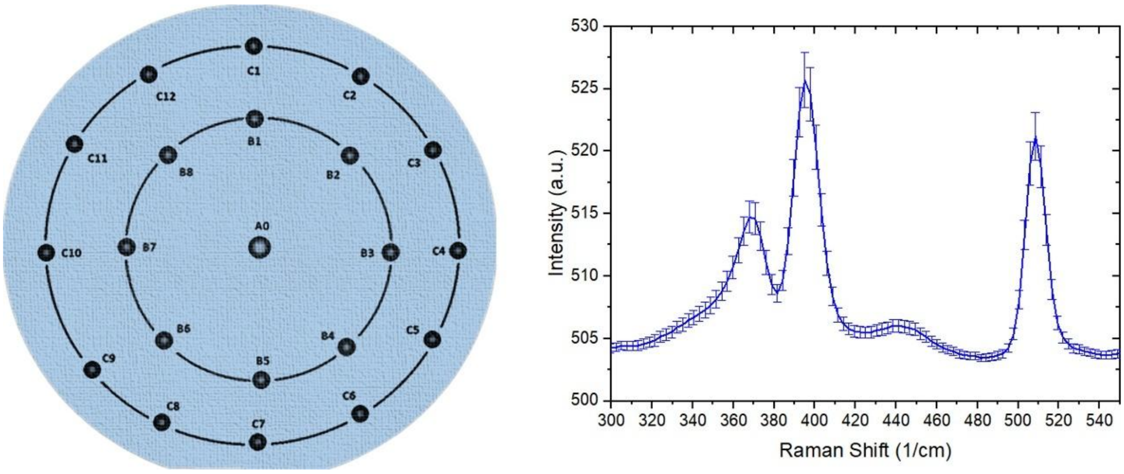

Figure S2. Raman spectra across the entire wafer for 40 ALD cycles. (Left) Spatial distribution of measurement points. (Right) Mean Raman spectrum with standard deviation.

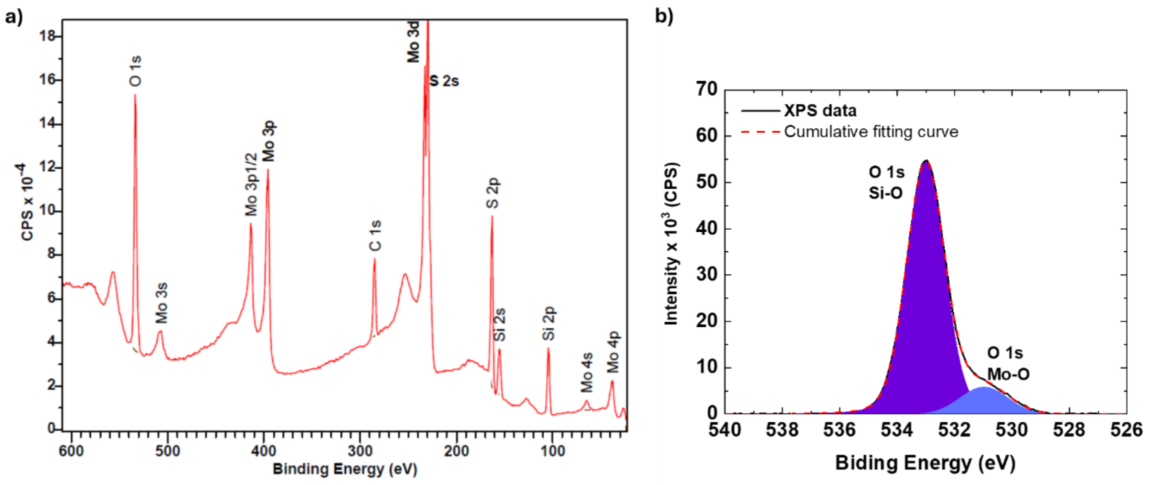

Figure S3. (a) Wide XPS spectrum for a MoS_2 sample. (b) High-resolution XPS spectrum of the O 1s core levels.

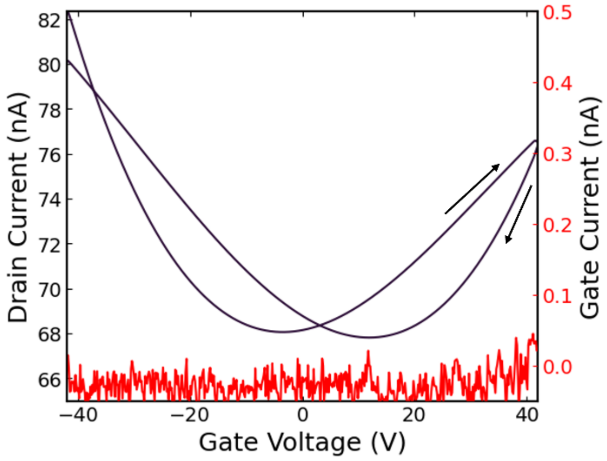

Figure S4. Transfer characteristics (I_D-V_G) and gate current for a device with L = 800 nm, demonstrating low leakage current in red.

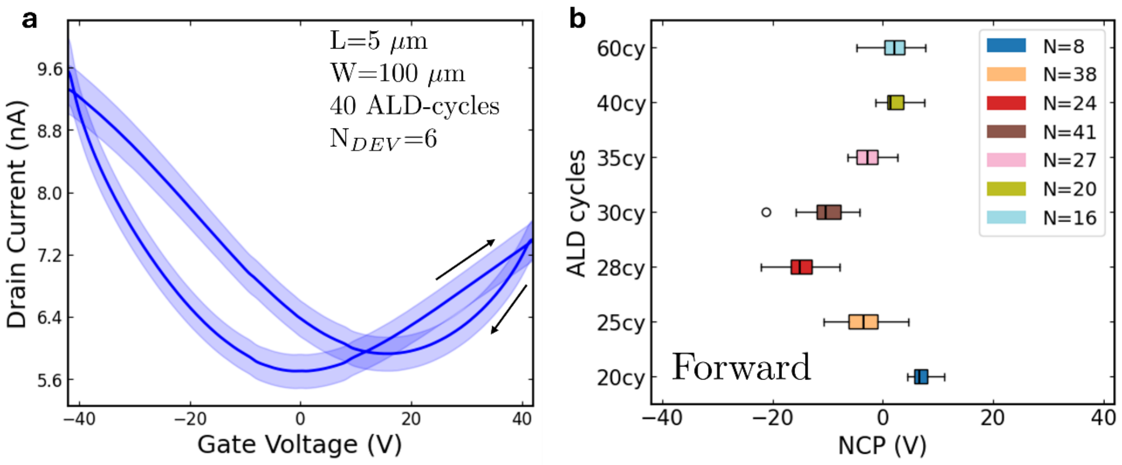

Figure S5. (a) Transfer characteristics of several devices with the same aspect ratio, fabricated with 40 ALD cycles. The average response (solid line) and standard deviation (shaded area) are shown for double-sweep measurements. (b) Boxplot of the Neutral Charge Point (NCP) extracted from devices of varying channel lengths, measured across the wafer for ALD thicknesses ranging from 20 to 60 cycles. N corresponds to the number of measured devices.

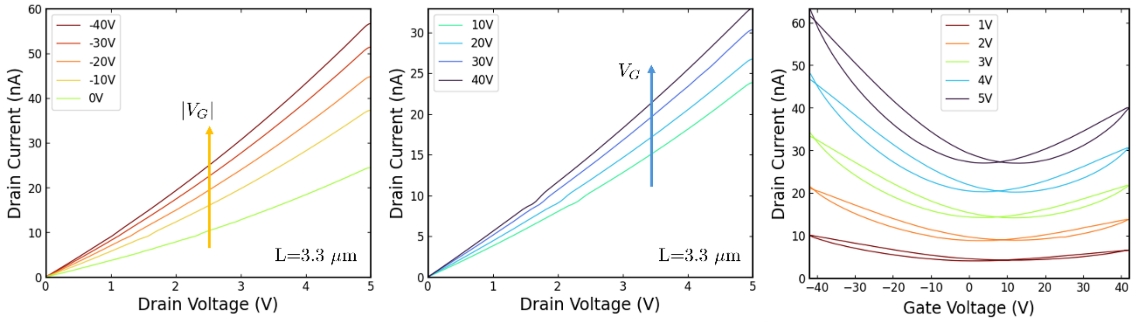

Figure S6. Output characteristics for (left) -40 to 0V and (middle) 0 to 40V. (Right) Transfer characteristics for V_D from 1 to 5 V.

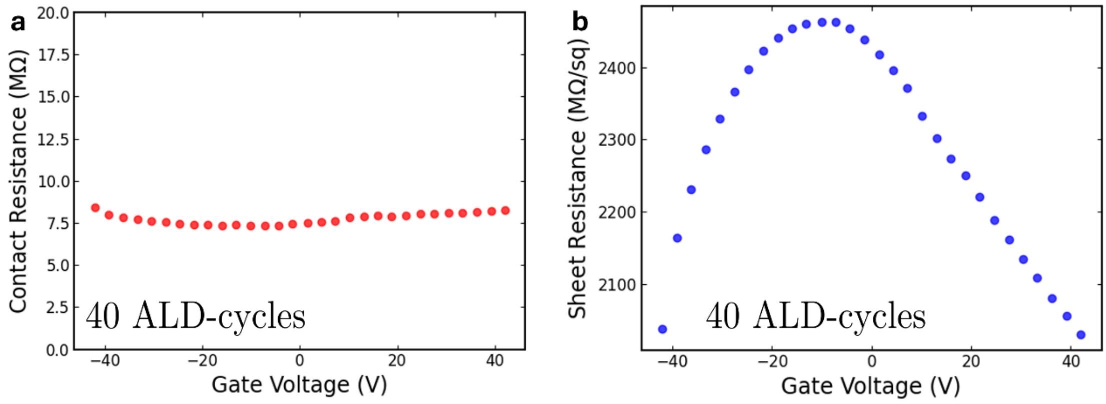

Figure S7. (a) Extrapolation of contact resistance (R_C) and (b) sheet resistance (R_{sh}) as function of the gate voltage.

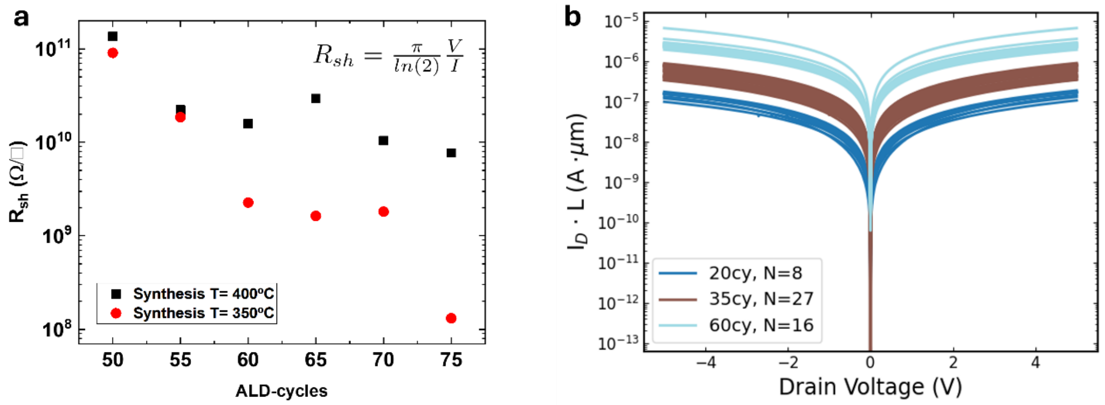

Figure S8. (a) Sheet resistance measured using the four-probe method for MoS_2 layers synthesized at different temperatures and ALD cycles. (b) Length-normalized drain current for devices with 20, 35, and 60 ALD cycles. N corresponds to the number of measured devices.

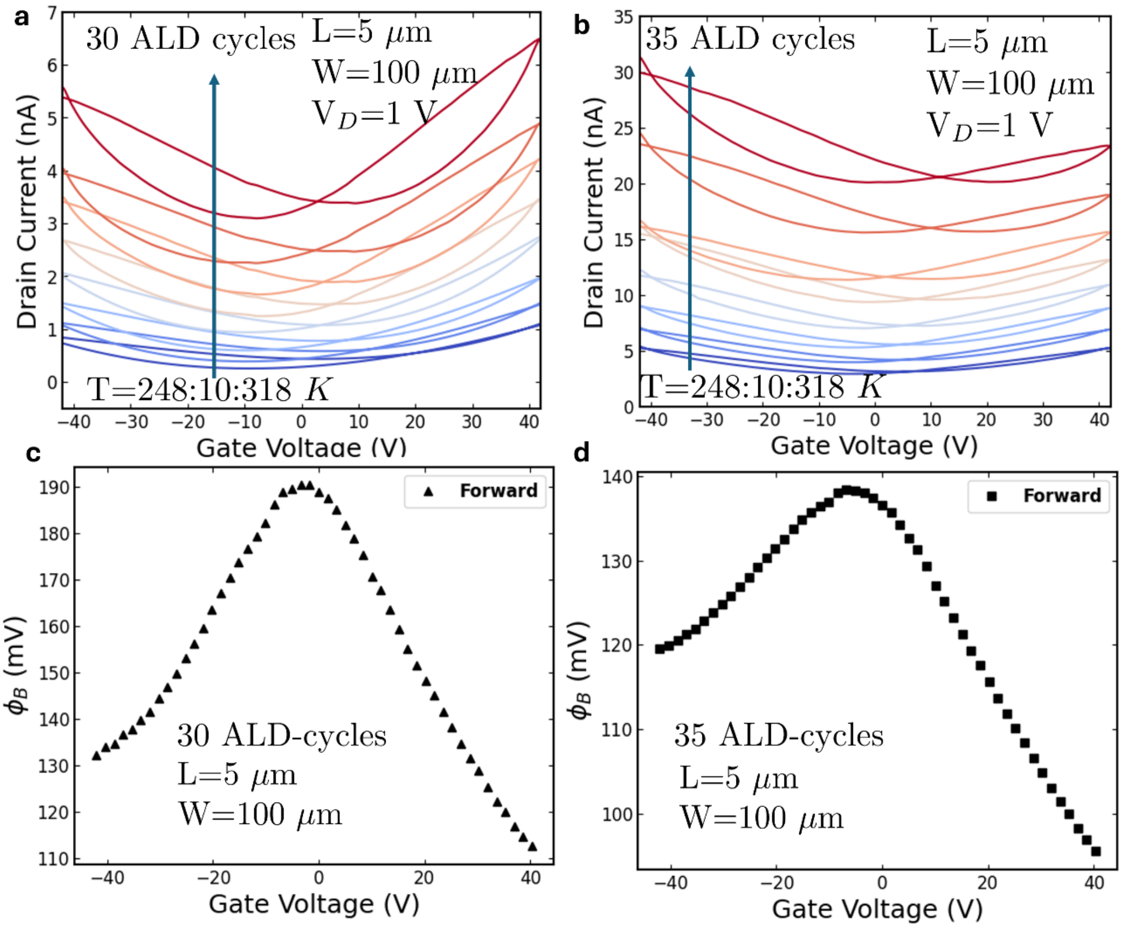

Figure S9. Transfer characteristics at different temperatures for 30 ALD cycles (a) and 35 ALD cycles (b). Extrapolated ϕ_B as a function of gate voltage for the case of 30 ALD cycles (c) and 35 ALD cycles (d).

Supplementary Note 1.

Due to the various potential mechanisms responsible for the absence of Fermi-level pinning and the low Schottky barrier heights, we have conducted an extensive characterization and analysis of the transport mechanisms in our devices to clarify this aspect:

- Temperature-dependent measurements indicate that thermionic emission is the dominant transport mechanism in our MoS_2 devices. The drain current (I_D) increases with temperature, and a linear relationship is observed in the Arrhenius plot (Figure 4a). This linearity confirms **thermally activated transport over a potential barrier**. Furthermore, the slope of the Arrhenius plot increases with the applied drain voltage, consistent with barrier lowering under higher electric fields, providing additional support for the thermionic emission model.

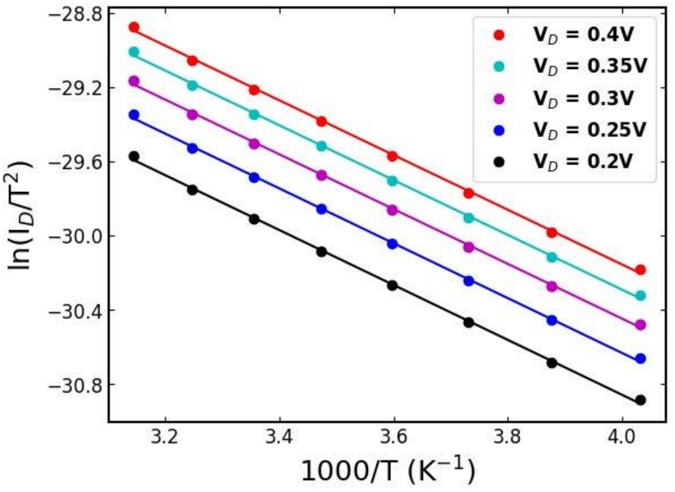

Figure S10: Arrhenius plot showing $\ln(I_D/T^2)$ versus 1000/T curves for various V_D values for a device with L = 0.85 µm and W = 100 µm.

Interestingly, our extracted Schottky barrier heights are relatively low for both electrons and holes, which is atypical for back-gated MoS_2 FETs and nearly symmetrical, as shown in Figure 4b. To gain insight into the origin of this behavior, we independently analyzed the temperature dependence of the contact resistance (R_c) and sheet resistance (R_{sh}) -or equivalently, the contact and sheet conductivities (σ)- of the devices. These two contributions were decoupled using the Transfer Length Method (TLM), allowing us to isolate the effects at the metal/semiconductor interface from those in the channel:

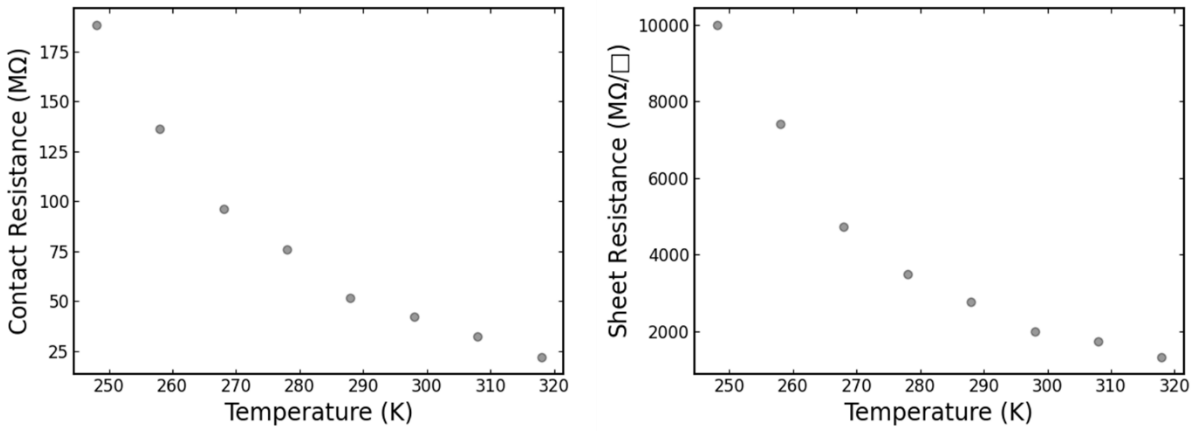

Figure S11: Contact (left) and Sheet (right) resistances as a function of the temperature for the same device.

We found that the material conductivity data fits an exponential expression of the form:

$$\sigma = \sigma_0 \, e^{\left[-\left(\frac{T_0}{T}\right)^n\right]} \qquad (1)$$

where n=1 and σ_0, T_0, and n are fitting parameters.

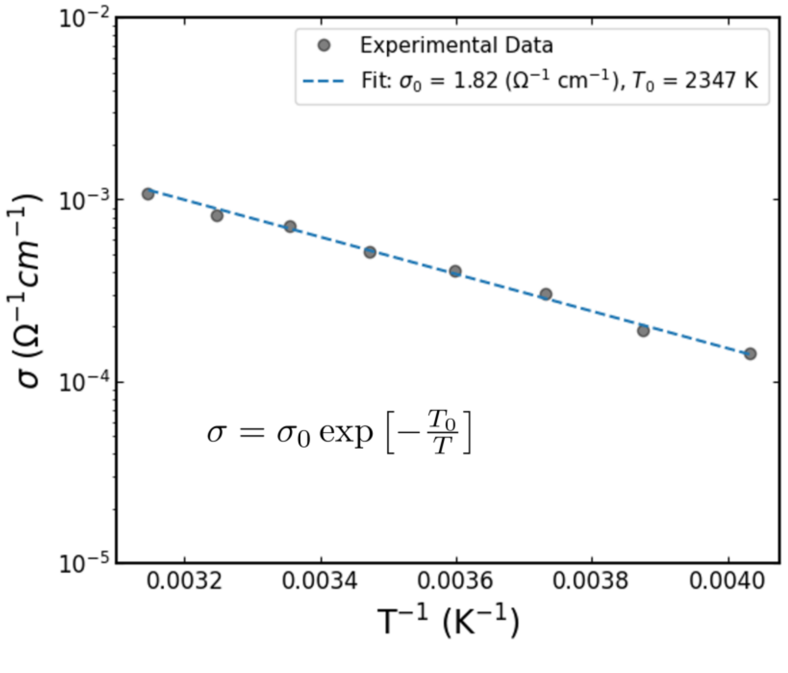

Figure S12: Material conductivity as a function of temperature.

The parameter that minimize the fit error are n=1.08, which is consistent with thermal activation and nearest neighbor hopping. This indicates that charge carriers require thermal energy to overcome localized states or potential barriers within the MoS_2 channel.

Referring to thermal activation, the obtained activation ($E_{act} = k_B T_0 \approx 200meV$) suggests that the Fermi level lies close to either the valence or conduction band. Similar value to the one observed in other ALD-grown TMDs[1]. Given the large bandgap of MoS_2 (~1.3 eV), such a position would typically limit ambipolar behavior and result in higher current levels due to easier

carrier injection. Therefore, the observed ambipolarity cannot be solely explained by simple thermal activation across the intrinsic bandgap, indicating that additional mechanisms influence the transport characteristics.

Hopping transport is typically associated with the presence of defects and localized states within the material. Although Raman analysis confirms the crystalline 2H phase of our MoS_2 samples, some degree of crystallographic misalignment is observed in GISAXS characterization. XPS measurements further indicate a stoichiometry below the ideal value of 2. This structural disorder can introduce localized states either at the band edges or within the bandgap, as described by Mott's model for dichalcogenides[2]. According to this model, despite maintaining a continuous density of states, spatially localized states arise due to disorder-induced tail states or mid-gap defect levels, which facilitate carrier hopping transport.

In this context, the observed nearest neighbor hopping (NNH) behavior, indicated by an exponent n = 1 in the conductivity temperature dependence, suggests that carrier transport occurs predominantly via hopping between spatially localized states. The relatively low values of σ_0 and $k_B T_0$ further support this interpretation, consistent with a transport mechanism dominated by defect-induced localized states rather than band-like conduction. Similarities have been observed in other works [3,4].

The field-effect mobility (μ_{FE}) increases with temperature from 250 K to 320 K, consistent with a thermally activated hopping transport mechanism. In this conduction process, higher temperatures provide charge carriers with the thermal energy needed to hop between localized states, leading to enhanced mobility.

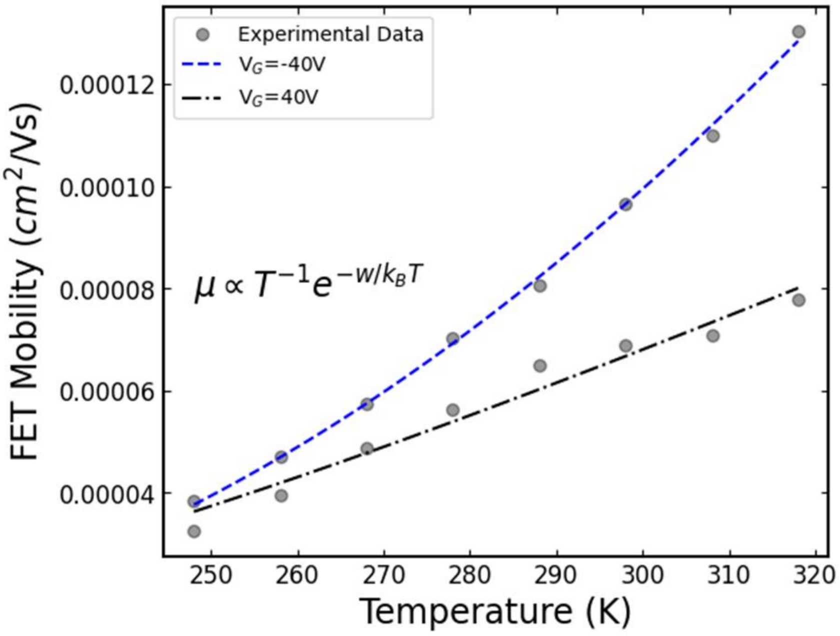

Figure S13: Field-Effect-Transistor mobility as a function of temperature for positive and negative gate voltages.

Contact/semiconductor junction

The thermionic emission model for the metal–semiconductor Schottky barrier fits the data well and enables approximate extraction of the barrier height, in good agreement with the more rigorous analysis presented in the manuscript[5].

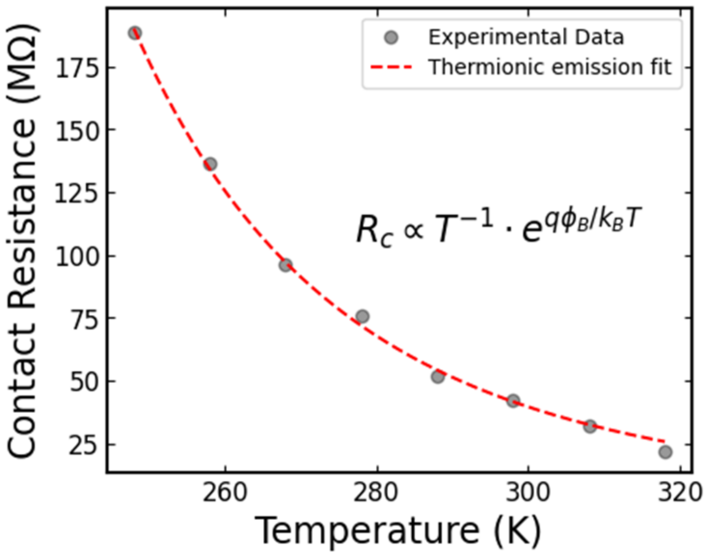

Figure S14: Contact resistance as a function of temperature.

The temperature dependence of the contact resistance (R_c) is also well described by this model. By fitting the thermionic emission equation, we extract Schottky barrier heights consistent with those obtained from comprehensive analyses elsewhere in this work. The observed decrease in contact resistance with increasing temperature supports a thermionic-diffusion transport regime at the metal–semiconductor interface.

This excellent fit of the contact resistance temperature dependence to the thermionic emission model supports thermally activated transport as the dominant mechanism at the metal–semiconductor interface. This observation, combined with the relatively low carrier density (~10^{13} cm^{-2}), allows us to reasonably exclude tunneling mechanisms such as direct or Fowler–Nordheim tunneling, which typically require higher carrier densities or thinner barriers. Furthermore, although band modulation effects induced by gate and drain biases can influence device behavior (as observed in recent works on WS_2 nanotubes[6]), they are unlikely to solely account for the observed ambipolarity given the transport signatures and temperature dependence observed.

Finally, the Poole–Frenkel (PF) emission model, which has been reported as the dominant transport mechanism in other low-dimensional systems such as ambipolar carbon nanotube devices, was also considered as a possible explanation. In such cases, temperature-dependent measurements typically deviate from thermionic emission behavior; specifically, follows this next equation:

$$J \propto n_0 V \exp\left[-\frac{q}{kT}\left(\Phi_B - \sqrt{\frac{qV}{\pi d \varepsilon_0 \varepsilon}}\right)\right] \quad (2)$$

Therefore, the PF model predicts a characteristic linear dependence of ln(I/V) on $V^{0.5}$, corresponding to field-enhanced emission from trap states, with the extracted trap barrier height often peaking near the charge neutrality point. However, in our MoS_2 devices, the current does not follow this expected trend. Plots of ln(I/V) versus $V^{0.5}$ fail to display linear behavior, as

illustrated in the data below. These observations suggest that Poole–Frenkel emission is unlikely to be the dominant transport mechanism in our devices.

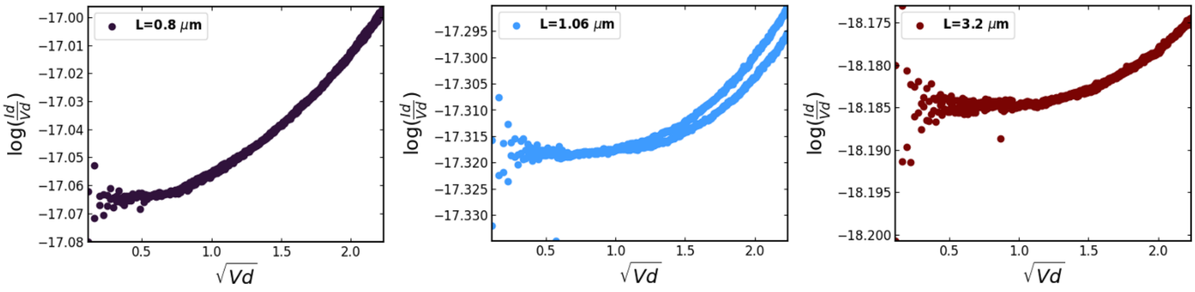

Figure S15: Log $\left[\frac{I_D}{V_D}\right]$ vs $\sqrt{V_D}$ for devices with three different channel lengths.

Conclusion

Temperature-dependent measurements clearly indicate that thermionic emission dominates through the semiconductor/metal interface in these devices. Concurrently, structural disorder in the MoS_2 channel—evidenced by XRD and XPS-confirmed sulfur and molybdenum vacancies—significantly influences the electronic properties. According to Mott's model, this disorder could introduce localized states near the band edges or within the bandgap, consistent with the observed thermally activated nearest-neighbor hopping conduction. These defects may also cause band tailing, softening the band edges and narrowing the gap between conduction and valence bands as the model proposed by Mott[2]:

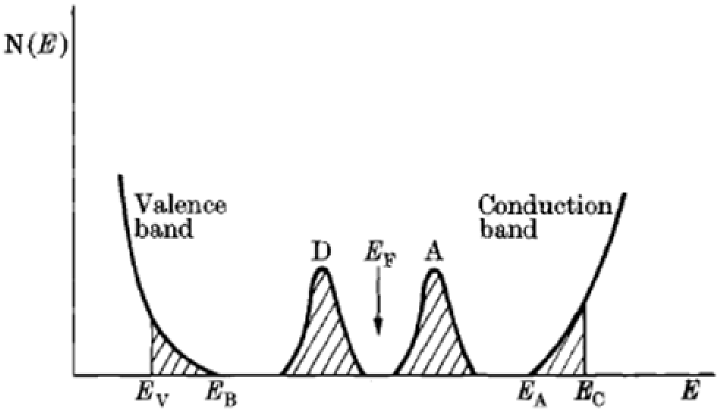

Figure S16: Model of density of states in material containing deep donors (D) below acceptors (A) proposed by Mott[9].

As a result, low and nearly symmetric Schottky barriers form at the metal–semiconductor interface may be expected. This enables balanced injection of both electrons and holes, leading to ambipolar behavior. Furthermore, alternative transport models such as Fowler–Nordheim tunneling and Poole–Frenkel emission can be discarded due to the relatively low carrier density (~10^{13} cm^{-2}), the observed temperature dependence, and the lack of fit to their characteristic signatures.

Strategies for Enhancing Performance:

- Defect Passivation: To reduce the impact of defects, we are exploring defect passivation techniques, such as surface treatments and annealing processes. We hypothesize that

annealing under sulfur conditions could help remove residual oxidation species (such as MOx) and improve the quality of the MoS_2 material.

- Material Thickness Optimization: We are considering the use of monolayer or few-layer MoS_2 in future device fabrication. Monolayer MoS_2 will provide better gate electrostatic control and reduce interlayer defects, leading to better on/off ratios and enhanced device performance.
- Gate Dielectric Engineering: We are also exploring the optimization of the gate dielectric material to improve short-channel control. This could contribute to better on/off ratios and make the devices more scalable for logic applications.

While the current on/off ratio is not yet ideal for practical applications, these strategies are already being implemented in our ongoing research. We are confident that these efforts will lead to significant improvements in device performance, making them more suitable for CMOS-compatible logic circuits in the near future.

Table S1. Benchmarking of Direct ALD-Grown Crystalline MoS_2: Material, Precursors, and FET Performance

**Carrier type-TMD**	**Precursors**	**Growth T. (°C)**	**Thickness (nm)**	**Annealing T(°C) /t(min) /atm**	**Capping layer**	**I_{ON} at $\vert V_{DS}\vert \approx$1V (nA/$\mu$m)**	**μ_{FE} (cm²/V·s)**	**ON-OFF Ratio (A/A)**	**Contact Resistance ($k\Omega \cdot \mu$m)**	**Sheet Resistance ($k\Omega \cdot \square$)**	**Ref.**
n-MoS_2	$Mo(NMe_2)_4$ and H_2S	80	–	1000/5/S	–	–	0.23	$\approx 10^2$	–	–	[7]
p-MoS_2	$Mo(CO)_6$ and Diethyl disulfide (DEDS)	250	5-6	450/30/Ar	30nm HfO_2	–	0.004	$\approx 10^3$	–	–	[8]
n-MoS_2	$Mo(CO)_6$ and DEDS with DES-inhibitor pretreatment	250	4-5	450/30/Ar	30nm HfO_2	–	13.9	$\approx 10^8$	–	–	[8]
n-MoS_2	$MoCl_5$ and H_2S	420	1.3-2.6	(930/0.5/Ar)	–	–	–	$\approx$ 100 (10000)	–	–	[9]
n-MoS_2	MoF_6 and H_2S	700	0.65	–	30nm Al_2O_3	30 (V_G=5 V)	0.1	$\approx 10^6$	–	–	[10]
n-MoS_2	$MoCl_5$ and $S(SiMe_3)_2$	375	5.6	800/45/Ar	Al_2O_3 front-gate	$\approx$ 25	3.53	$\approx$ 100	–	–	[11]
n-MoS_2	$MoCl_5$ and hexamethyl-disilathiane (HMDST)	350	2.9	900/30-120/S	25 nm Al_2O_3 front-gate	<60	0.56 to 9.83	$\approx 10^6$	–	–	[12]
n-MoS_2	$MoCl_5$ and HMDST	300	2.7	900/45'/S	25 nm Al_2O_3 front-gate	$\approx$ 20	3	$\approx$ 1000	–	–	[13]
n-MoS_2	$Mo(CO)_6$ and HMDST	150	3.09	800/–/S	25 nm Al_2O_3 front-gate	$\approx$ 100	3	$\approx 10^3$	143	57	[14]
n-MoS_2	$MoCl_5$ and H_2S	900	0.8	–	40nm Al_2O_3 front-gate	10^{-6} (A)	0.2	$\approx 10^8$	–	–	[15]
n-MoS_2	$MoCl_5$ and H_2S	390	9	–	–	0.0004 (V_{DS} $\approx$0.5V)	1	$\approx 10^3$	–	–	[16]
MoS_2	$Mo(N^tBu)_2$-$(NMe_2)_2$ and H_2S	100	18.5	–	–	0.2	$\approx 10^{-3}$ (Hall)	$\approx$ 5	–	90	[17]
MoS_2	$Mo(N^tBu)_2$-$(NMe_2)_2$ and H_2S	350	10.6	–	–	0.2	$\approx 7\cdot 10^{-2}$ (Hall)	$\approx$ 5	–	$22.6\cdot 10^3$	[17]
MoS_2	$Mo(N^tBu)_2$-$(NMe_2)_2$ and di-tert-butyl disulfide (TBDS)	350	3	–	–	–	–	–	–	$4.5\cdot 10^4$-$5.2\cdot 10^4$	[18]
ambipolar-MoS_2	$Mo(N^tBu)_2$-$(NMe_2)_2$ and H_2S	400	5-7	–	–	1 (V_G=-40 V)	$\approx 2\cdot 10^{-4}$	$\approx$ 2	$3\cdot 10^3$	10^6	This work

Table S2. Benchmarking of Direct ALD-Grown Crystalline WS_2: Material, Precursors, and FET Performance

**Carrier type-TMD**	**Precursors**	**Growth T. (^oC)**	**Thickness (nm)**	**Annealing T(^oC) /t(min) /atm**	**Capping layer**	**I_{ON} at $|V_{DS}|\approx$1V (nA/μm)**	**μ_{FE} (cm^2/V·s)**	**ON-OFF Ratio (A/A)**	**Contact Resistance ($k\Omega \cdot \mu m$)**	**Sheet Resistance ($k\Omega \cdot \square$)**	**Ref.**
n-WS_2	WCl_6 and HMDST	400	5.6	950/120/S	30nm Al_2O_3 front-gate	100	3.21 (10.55 Hall)	$\approx 10^5$	–	≈5.5	[19]
n-WS_2	WCl_6 and HMDST	400	5.6	950/120/S	20nm Al_2O_3 front-gate	100	0.32-6.85 (86.3 Hall)	$\approx 10^5$	–	≈5.5	[20]
p-WS_2	WF_6 and H_2S	300	≈ 2.6	900/2/Inert	Al_2O_3	≈ 1 ($V_G \approx$-2.5 V)	–	8000	5500	168000	[21]
n-WS_2	WCl_5 and H_2S	390	2.5	–	–	–	12	$\approx 10^4$	–	–	[22]
p-WS_2	$W(N^tBu)_2$-$(NMe_2)_2$ and H_2S	450	8	–	–	–	23.2 (Hall)	–	26.6·10^3 $R_cW=\rho_c/L_T$	1.58·10^4	[1]
p-WS_2	WF_6 and H_2S	450	2.0-2.6	–	Al_2O_3	10 ($V_G \approx$-25 V)	–	$\approx 10^5$	–	–	[23]
ambipolar-WS_2	WF_6 and H_2S	–	–	–	Al_2O_3 front-gate	<1	–	≈ 10	–	–	[24]
WS_2	–	450	–	–	Al_2O_3 front-gate	<1 (V_G=-1 V/nm)	–	>10^4	–	–	[25]

Supplementary Note 2.

Low frequency noise characterization, as shown in Figures 6c and 6d of the manuscript, can be performed in order to get the interface trap state density [7,8]. As discussed, data do not conform to the mobility fluctuation model (Hooge model), which predicts an inverse dependence of the power spectral density (PSD) on current. In our measurements, the PSD shows no significant dependence on gate voltage. Similarly, although the frequency spectra in Figure 6c exhibit a clear 1/f behavior, the results are inconsistent with the carrier number fluctuation model (McWhorter model), which would typically show a correlation between the normalized noise power and the normalized transconductance (Figure 6d).

The model that combines carrier number fluctuations with correlated mobility fluctuations—commonly referred to as the carrier number with correlated mobility fluctuations model (CNF-CMF)[9], may offer a more accurate description of the observed noise behavior. This model accounts for the interplay between trap-induced carrier number fluctuations and their correlated effect on mobility, leading to a gate-voltage-dependent normalized power spectral density. In regimes of moderate and strong inversion, the model is typically described by the following expression:

$$\frac{S_{ID}}{I_D^2} = S_{VG}\left(\frac{g_m}{I_D}\right)^2 = S_{Vfb}\left(1 + \Omega\frac{I_D}{g_m}\right)^2\left(\frac{g_m}{I_D}\right)^2 \tag{3}$$

where $\Omega = \alpha_{sc}\mu_{eff}C_{ox}$ with α_{sc} representing the Coulomb scattering coefficient, μ_{eff} the effective mobility, and C_{ox} the oxide capacitance. The term S_{Vfb} denotes the flat-band voltage spectral density, whose expression depends on the specific trapping mechanism within the oxide. While direct temperature-dependent noise measurements were not performed, the thermally activated behavior observed in contact and sheet resistance suggests that similar mechanisms—such as hopping or defect-mediated conduction—may influence trap dynamics. Based on this, we adopt a thermally activated model for oxide trapping consistent with prior studies. In the case of a thermally activated trapping process, the flat-band voltage spectral density can be expressed as[10]:

$$S_{Vfb} = \frac{q^2kT^2N_{it}}{WLC_{ox}^2f\Delta E_a} \tag{4}$$

where ΔE_a is the amplitude of the activation energy dispersion and N_{it} is the trap surface state density (eV^{-1} cm^{-2}).

As can be seen from Figure S17, the CNF-CMF model provides a reasonable fit to the measured drain current noise,

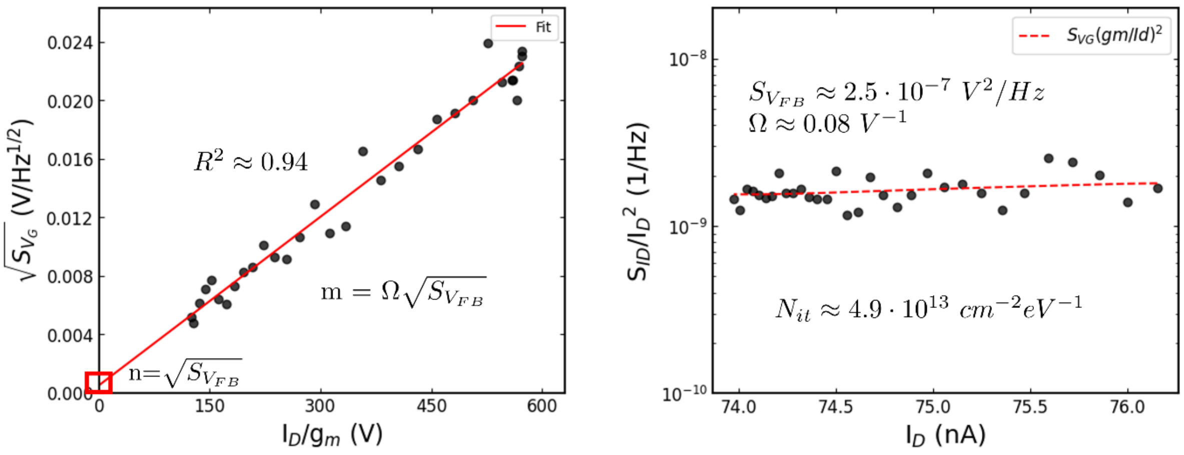

Figure S17: (Left) Square root of the input gate voltage noise $\sqrt{S_{VG}}$ as a function of Id/gm for a device. (Right) Normalized current noise spectral densities (measured in solid and modelled according to the CNF-CMF approach in dashed line) as a function of the drain current. Frequency f = 10 Hz. T = 300 K.

Based on this agreement, we can estimate the oxide trap surface state density. Assuming a thermal activation energy of 0.2 eV — a value consistent with the temperature dependence observed in both contact and sheet resistance — the extracted trap density is on the order of 10^{13} $eV^{-1}cm^{-2}$, which is consistent with values reported for MoS_2/amorphous oxide interfaces [11,12].
Moreover, the interface trap density can be estimated from the hysteresis in the transfer characteristics, using the relation $N_t = \frac{C_{ox}\Delta V_{th}}{q}$. For devices with 40 ALD cycles, this yields a value $> 10^{12}$ cm^{-2}, in reasonable agreement with the extracted values using low-frequency noise characterization.

Moreover, as noted in the manuscript, the gate-voltage independence of the PSD also points to a contact-dominated noise regime. This occurs when both the contact resistance and the associated noise amplitude are comparable to or exceed the contributions from the channel. While some studies attribute this flat PSD behavior entirely to the MoS_2/oxide interface [8], others have observed a transition from contact-dominated to channel-dominated noise as a function of gate bias in TMD-based FETs [13], allowing for a clear distinction between the two regimes. In our case, no such transition is observed, likely due to limited current modulation, which hinders the decoupling of contact and channel noise components. However, similar behavior is observed even in devices with different channel lengths, including those where the contact contribution is notably smaller (see Figure below). Since the extracted activation energy remains consistent with channel-dominated conduction processes, the estimated trap density remains a valid representation of the oxide interface quality.

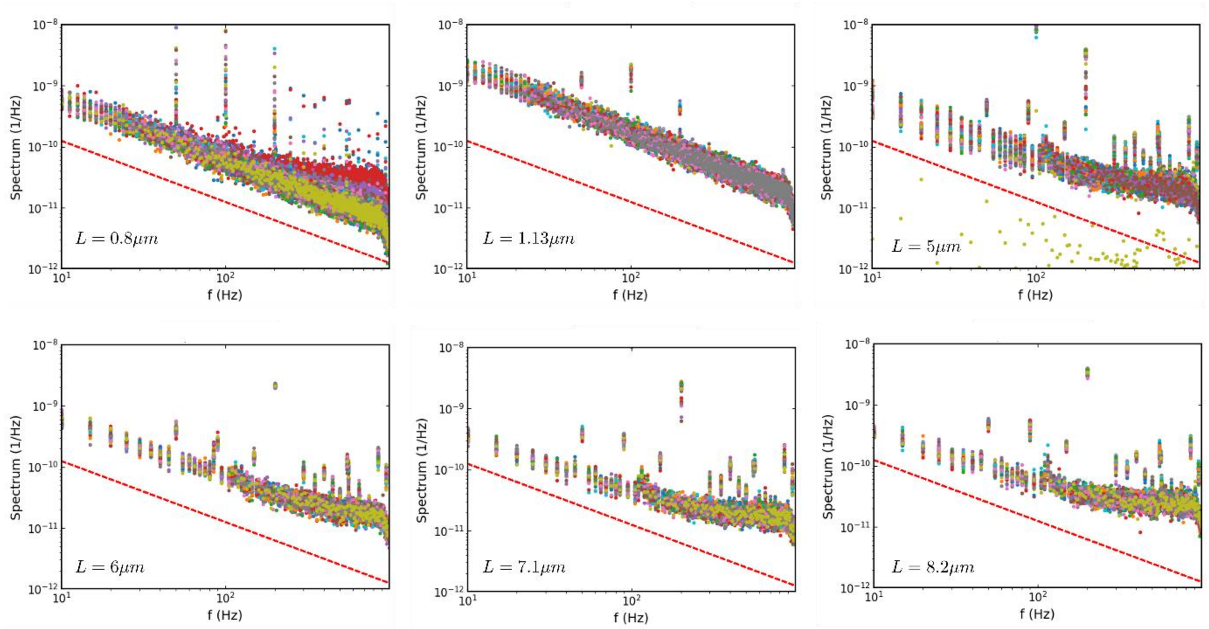

Figure S18: Normalized current noise spectral densities as a function of the frequency for devices with different channel lengths. V_D=2 V V_G=-5:0.25:5 V.

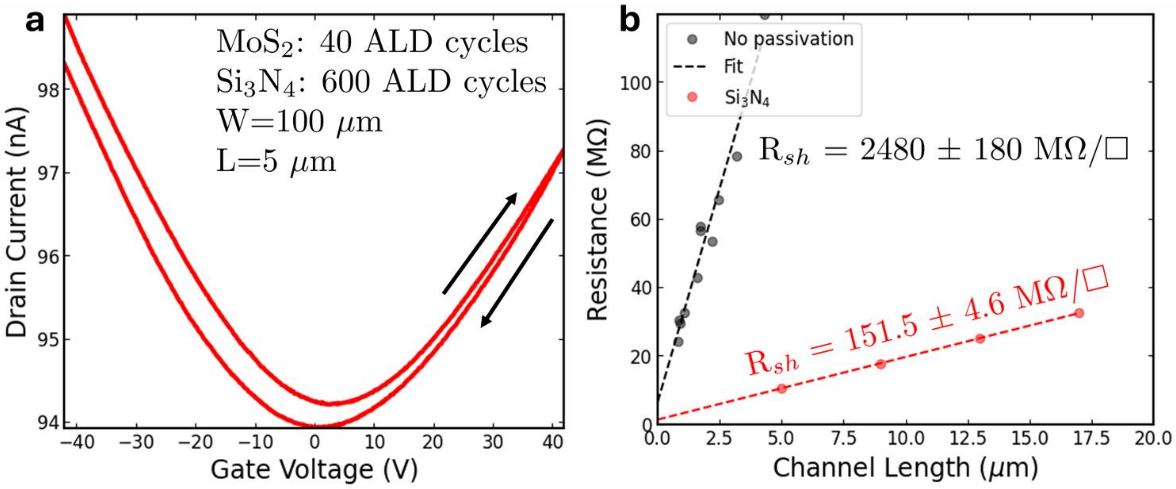

Figure S19. (a) Transfer characteristics of an ALD-grown MoS_2 device passivated with Si_3N_4, deposited without vacuum break between synthesis and encapsulation. (b) Extracted sheet resistance for passivated and unpassivated devices, showing enhanced conductivity with dielectric encapsulation.

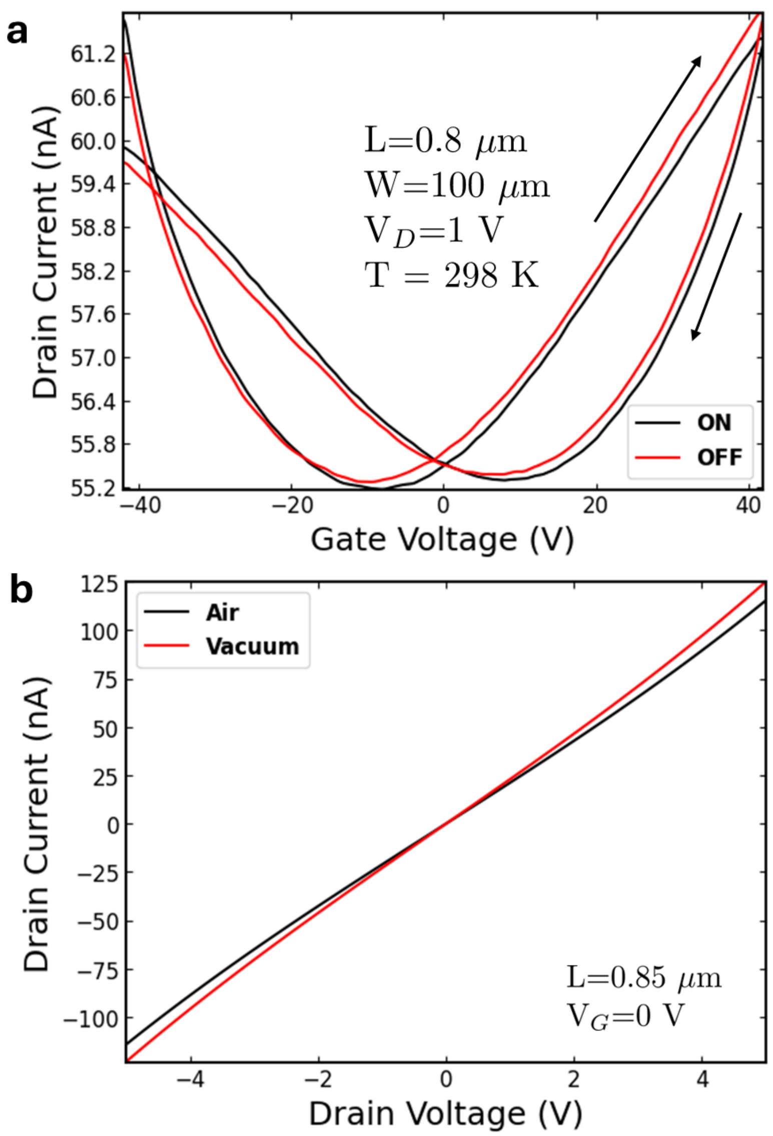

Figure S20. a) Transfer characteristics of an 800 nm-length device under artificial light (ON) and dark ambient (OFF) conditions. b) Output characteristics of the device measured in air and under vacuum (10^{-5}mbar) conditions.

References

[1] Coleman, E.; Monaghan, S.; Gity, F.; Mirabelli, G.; Duffy, R.; Sheehan, B.; Balasubramanyam, S.; Bol, A. A.; Hurley, P. *Applied Physics Letters* **2023**, *123*, 011901.

[2] Mott, N. F.; Davis, E. A. *Electronic processes in non-crystalline materials*, 2nd ed.; International series of monographs on physics; Clarendon press: Oxford, 2012.

[3] Ghatak, S.; Pal, A. N.; Ghosh, A. *ACS Nano* **2011**, *5*, 7707–7712.

[4] Campbell, P. M.; Perini, C. J.; Chiu, J.; Gupta, A.; Ray, H. S.; Chen, H.; Wenzel, K.; Snyder, E.; Wagner, B. K.; Ready, J.; Vogel, E. M. *2D Materials* **2017**, *5*, 015005.

[5] Schroder, D. K. *Semiconductor Material and Device Characterization: Third Edition*, 3rd ed.; John Wiley & Sons, 2005; pp 1–779.

[6] Pelella, A.; Camilli, L.; Giubileo, F.; Zak, A.; Passacantando, M.; Guo, Y.; Intonti, K.; Kumar, A.; Bartolomeo, A. D. *Nanoscale* **2025**, *17*, 2052–2060.

[7] Jurca, T.; Moody, M. J.; Henning, A.; Emery, J. D.; Wang, B.; Tan, J. M.; Lohr, T. L.; Lauhon, L. J.; Marks, T. J. *Angew. Chem., Int. Ed.* **2017**, *56*, 4991.

[8] Jeon, W.; Cho, Y.; Jo, S.; Ahn, J.-H.; Jeong, S.-J. *Adv. Mater.* **2017**, *29*, 1703031.

[9] Ahn, W.; Lee, H.; Cho, Y.; Byun, K.-E.; Kim, H.; Leem, M.; Lee, H.; Park, T.; Lee, E.; Shin, H.-J.; Kim, H. *physica status solidi (a)* **2020**, *217*.

[10] Kim, Y.; Choi, D.; Woo, W. J.; Lee, J. B.; Ryu, G. H.; Lim, J. H.; Lee, S.; Lee, Z.; Im, S.; Ahn, J.-H.; Kim, W.-H.; Park, J.; Kim, H. *Applied Surface Science* **2019**, *494*, 591–599.

[11] Wang, Y.; Gu, Z.-H.; Liu, H.; Chen, L.; Liu, X.-k.; Min, L.; Li, Z.-w.; Zhu, H.; Sun, Q.-Q. *ACS Applied Electronic Materials* **2019**, *1*, 1418–1423, Publisher: American Chemical Society.

[12] Liu, H.; Chen, L.; Zhu, H.; Sun, Q.-Q.; Ding, S.-J.; Zhou, P.; Zhang, D. W. *Nano Research* **2020**, *13*, 1644–1650.

[13] Zhang, T.; Liu, H.; Wang, Y.; Zhu, H.; Chen, L.; Sun, Q.; Zhang, D. W. *physica status solidi (RRL) – Rapid Research Letters* **2019**, *13*.

[14] Zhang, T.; Wang, Y.; Xu, J.; Chen, L.; Zhu, H.; Sun, Q.; Ding, S.; Zhang, D. W. *2D Materials* **2017**, *5*, 015028.

[15] Kim, Y.; Song, J.-G.; Park, Y. J.; Ryu, G. H.; Lee, S. J.; Kim, J. S.; Jeon, P. J.; Lee, C. W.; Woo, W. J.; Choi, T.; Jung, H.; Lee, H.-B.-R.; Myoung, J.-M.; Im, S.; Lee, Z.; Ahn, J.-H.; Park, J.; Kim, H. *Scientific Reports* **2016**, *6*, 18754.

[16] Browning, R.; Padigi, P.; Solanki, R.; Tweet, D. J.; Schuele, P.; Evans, D. *Materials Research Express* **2015**, *2*, 035006.

[17] Mattinen, M.; Gity, F.; Coleman, E.; Vonk, J. F.; Verheijen, M. A.; Duffy, R.; Kessels, W. M.; Bol, A. A. *Chemistry of Materials* **2022**, *34*, 7280–7292.

[18] Campbell, I. E.; Gupta, A.; Metaxa, P.; Arifutzzaman, A.; Ma, T.; Arellano, P.; Duffy, R.; Bol, A. A. *Chemistry of Materials* **2025**, *37*, 1478–1490, Publisher: American Chemical Society.

[19] Yang, H.; Wang, Y.; Zou, X.; Bai, R.-X.; Han, S.; Wu, Z.; Han, Q.; Zhang, Y.; Zhu, H.; Chen, L.; Lu, X.; Sun, Q.; Lee, J. C.; Yu, E. T.; Akinwande, D.; Ji, L. *ACS Applied Materials & Interfaces* **2021**, *13*, 43115–43122.

[20] Yang, H.; Wang, Y.; Zou, X.; Bai, R.; Wu, Z.; Han, S.; Chen, T.; Hu, S.; Zhu, H.; Chen, L.; Zhang, D. W.; Lee, J. C.; Lu, X.; Zhou, P.; Sun, Q.; Yu, E. T.; Akinwande, D.; Ji, L. *Research* **2021**, *2021*.

[21] Schram, T.; Smets, Q.; Groven, B.; Heyne, M. H.; Kunnen, E.; Thiam, A.; Devriendt, K.; Delabie, A.; Lin, D.; Lux, M.; Chiappe, D.; Asselberghs, I.; Brus, S.; Huyghebaert, C.; Sayan, S.; Juncker, A.; Caymax, M.; Radu, I. P. WS_2 transistors on 300 mm wafers with BEOL compatibility. 2017 47th European Solid-State Device Research Conference (ESSDERC). 2017; pp 212–215.

[22] Browning, R.; Plachinda, P.; Padigi, P.; Solanki, R.; Rouvimov, S. *Nanoscale* **2016**, *8*, 2143–2148.

[23] Groven, B.; Nalin Mehta, A.; Bender, H.; Meersschaut, J.; Nuytten, T.; Verdonck, P.; Conard, T.; Smets, Q.; Schram, T.; Schoenaers, B.; Stesmans, A.; Afanasev, V.; Vandervorst, W.; Heyns, M.; Caymax, M.; Radu, I.; Delabie, A. *Chemistry of Materials* **2018**, *30*, 7648–7663.

[24] Huyghebaert, C.; Schram, T.; Smets, Q.; Kumar Agarwal, T.; Verreck, D.; Brems, S.; Phommahaxay, A.; Chiappe, D.; El Kazzi, S.; Lockhart de la Rosa, C.; Arutchelvan, G.; Cott, D.; Ludwig, J.; Gaur, A.; Sutar, S.; Leonhardt, A.; Marinov, D.; Lin, D.; Caymax, M.; Asselberghs, I.; Pourtois, G.; Radu, I. 2D materials: roadmap to CMOS integration. 2018 IEEE International Electron Devices Meeting (IEDM). 2018; pp 22.1.1–22.1.4.

[25] Asselberghs, I.; Smets, Q.; Schram, T.; Groven, B.; Verreck, D.; Afzalian, A.; Arutchelvan, G.; Gaur, A.; Cott, D.; Maurice, T.; Brems, S.; Kennes, K.; Phommahaxay, A.; Dupuy, E.; Radisic, D.; De Marneffe, J.-F.; Thiam, A.; Li, W.; Devriendt, K.; Huyghebaert, C.; Lin, D.; Caymax, M.; Morin, P.; Radu, I. Wafer-scale integration of double gated WS_2 -transistors in 300mm Si CMOS fab. 2020 IEEE International Electron Devices Meeting (IEDM). 2020; pp 40.2.1–40.2.4.

[26] Marquez, C.; Navarro, C.; Karg, S.; Ortega, R.; Zota, C.; Gamiz, F. *IEEE Transactions on Electron Devices* **2024**, *71*, 3964–3969.

[27] Vu, Q. A.; Fan, S.; Lee, S. H.; Joo, M.-K.; Yu, W. J.; Lee, Y. H. *2D Materials* **2018**, *5*, 031001.

[28] Ghibaudo, G.; Roux, O.; Nguyen-Duc, C.; Balestra, F.; Brini, J. *physica status solidi (a)* **1991**, *124*, 571–581.

[29] Jomaah, J.; Balestra, F.; Ghibaudo, G. *Journal of telecommunications and information technology* **2005**, *1*, 24–32.

[30] Takenaka, M.; Ozawa, Y.; Han, J.; Takagi, S. *Technical Digest - International Electron Devices Meeting, IEDM* **2017**, 5.8.1–5.8.4.

[31] Knobloch, T. On the Electrical Stability of 2D Material-Based Field-Effect Transistors. Ph.D. thesis, TU Wien, Wien, 2021.

[32] Kwon, J.; Prakash, A.; Das, S. R.; Janes, D. B. *Physical Review Applied* **2018**, *10*, 064029.
